# Research progress on the clinical application and mechanism of iguratimod in the treatment of autoimmune diseases and rheumatic diseases

**DOI:** 10.3389/fimmu.2023.1150661

**Published:** 2023-09-21

**Authors:** Zhiyong Long, Liuting Zeng, Qi He, Kailin Yang, Wang Xiang, Xiang Ren, Ying Deng, Hua Chen

**Affiliations:** ^1^ Department of Rehabilitation Medicine, Guangzhou Panyu Central Hospital, Guangzhou, China; ^2^ Department of Rheumatology and Immunology, Nanjing Drum Tower Hospital, Chinese Academy of Medical Sciences & Peking Union Medical College, Graduate School of Peking Union Medical College, Nanjing, China; ^3^ People's Hospital of Ningxiang City, Ningxiang, China; ^4^ Key Laboratory of Hunan Province for Integrated Traditional Chinese and Western Medicine on Prevention and Treatment of Cardio-Cerebral Diseases, School of Integrated Chinese and Western Medicine, Hunan University of Chinese Medicine, Changsha, China; ^5^ Department of Rheumatology, The First People's Hospital Changde City, Changde, Hunan, China

**Keywords:** iguratimod, autoimmune disease, rheumatic skeletal disease, arthritis, Sjogren’s syndrome, IgG4-RD, interstitial lung disease

## Abstract

Autoimmune diseases are affected by complex pathophysiology involving multiple cell types, cytokines, antibodies and mimicking factors. Different drugs are used to improve these autoimmune responses, including nonsteroidal anti-inflammatory drugs (NSAIDs), corticosteroids, antibodies, and small molecule drugs (DMARDs), which are prevalent clinically in the treatment of rheumatoid arthritis (RA), etc. However, low cost-effectiveness, reduced efficacy, adverse effects, and patient non-response are unattractive factors driving the development of new drugs such as iguratimod. As a new disease-modifying antirheumatic drug, iguratimod has pharmacological activities such as regulating autoimmune disorders, inflammatory cytokines, regulating immune cell activation, differentiation and proliferation, improving bone metabolism, and inhibiting fibrosis. In recent years, clinical studies have found that iguratimod is effective in the treatment of RA, SLE, IGG4-RD, Sjogren ‘s syndrome, ankylosing spondylitis, interstitial lung disease, and other autoimmune diseases and rheumatic diseases. The amount of basic and clinical research on other autoimmune diseases is also increasing. Therefore, this review systematically reviews the latest relevant literature in recent years, reviews the research results in recent years, and summarizes the research progress of iguratimod in the treatment of related diseases. This review highlights the role of iguratimod in the protection of autoimmune and rheumatic bone and related immune diseases. It is believed that iguratimod’s unique mode of action and its favorable patient response compared to other DMARDs make it a suitable antirheumatic and bone protective agent in the future.

## 1 Introduction

Autoimmune diseases and rheumatic skeletal diseases are pathological autoimmune reactions caused by multiple factors in the body. The diseases caused by the destruction and damage of self-tissues and cellular components through autoimmune reactions, resulting in tissue damage and organ dysfunction, can be divided into organ-specific autoimmune diseases and systemic autoimmune diseases (1, 2). Such diseases usually involve various systems of the body, such as blood, joints, muscles, bones, and soft tissues around joints. After diagnosis, patients should be treated in time to avoid damage to tissues, organs or systems caused by the further development of the disease (3). Epidemiological data show that the pathogenesis of autoimmune diseases has not been fully clarified, but it has some obvious disease characteristics: (1) The etiology is mostly unknown, spontaneous or idiopathic, and some are related to bacterial, viral infection or taking certain drugs; (2) There are more women than men; (3) The course of the disease is repeated, showing a process of chronic migration; (4) There is an obvious family tendency; (5) Disease overlap phenomenon, that is, a patient can suffer from two or more than two kinds of AID at the same time (4–6). The pathological features of AID are mainly caused by abnormal autoimmune response, usually involving joints, muscles, bones and surrounding soft tissues (7–9). At present, the main drugs for the treatment of autoimmune diseases and rheumatic bone diseases mainly include non-steroidal anti-inflammatory drugs (NSAIDs) (10, 11), glucocorticoids (12), traditional disease-modifying antirheumatic drugs (DMARDs) (13, 14), herbal preparations (15, 16), biological preparations DMARDs, etc. (17, 18). For a wide range of highly heterogeneous autoimmune diseases and rheumatic bone diseases, the research and development of new drugs is urgently needed.

**Figure 1:**
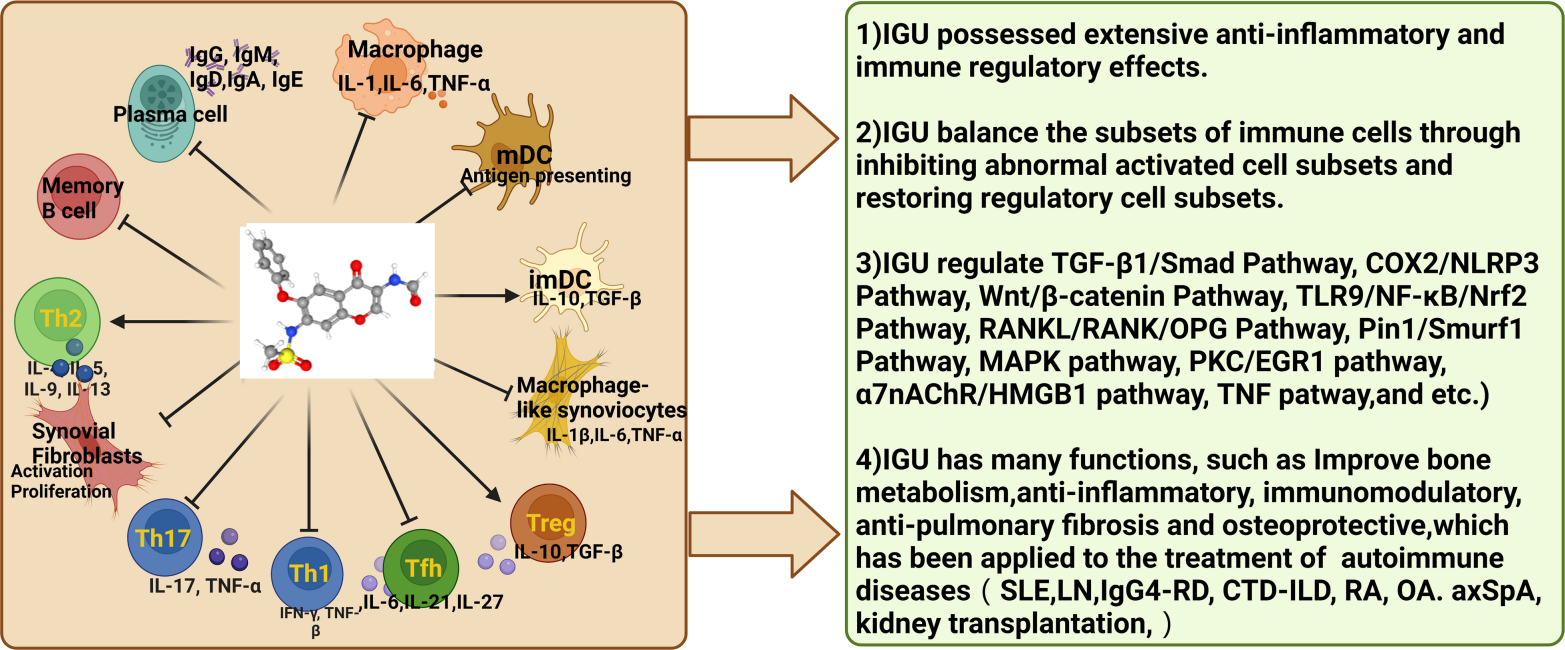
Regulatory mechanism of Iguratimod on immune cells (Iguratimod has been found to modulate immune cell function and activity to balance immune cell subsets, thereby further reducing inflammation and tissue damage in autoimmune diseases. mDCs, mature DCs; imDCs, immature dendritic cells.).

Iguratimod is a new type of DMARDs, which is a derivative of oxonetone containing diamide, formyl and toluenesulfonamide functional groups (19). Iguratimod was approved for marketing in China in 2011, and it is currently widely used as a new type of small molecule DMARDs in the treatment of RA and other autoimmune diseases and rheumatic bone diseases (20). A number of studies have shown that in the treatment of RA, iguratimod not only has immunomodulatory and anti-inflammatory effects, but also has a unique bone protection effect (21). In the treatment of RA, most adverse reactions can be relieved or disappeared after drug reduction or withdrawal (22). No serious adverse reactions have been found so far, and patients are well tolerated. Compared with traditional DMARDs such as methotrexate (MTX) and leflunomide, it has fewer side effects and higher safety (23). And clinical trials have shown that the combination of iguratimod and MTX is safe and effective in the treatment of RA, and the effect is stronger than that of monotherapy. In addition to the traditional treatment of RA, Iguratimod is also used in pSS, SLE, immune-related interstitial lung disease, IgG4-RD, ankylosing spondylitis, bone destruction in multiple myeloma (MM), and osteoarthritis (OA), organ transplantation and transplant rejection immunity (kidney transplantation) and other immune-related diseases (24, 25). This review summarized the latest research progress of iguratimod, and expounds its related mechanism, laying the foundation for further research. The regulatory mechanism of Iguratimod on immune cells were shown in **Figure 1**.

## 2 Iguratimod: a chemical substance with multiple pharmacological activities

### 2.1 Mechanism of iguratimod regulating immune cells

The pathogenesis of autoimmune diseases and rheumatic skeletal diseases (such as rheumatoid arthritis) mainly includes the breakdown of immune tolerance, which is closely related to the interaction of genetic factors, infection and other environmental factors. Autoimmunity can be defined as an adaptive immune response specific to self-antigens, whereas autoantibodies are antibodies directed against normal cellular components that elicit a response called self-antigens (26–28). This evolution process is mainly divided into multiple stages: congenital or acquired triggers, genetic predisposition and environmental factors may lead to the loss of autoimmune tolerance, and under the influence of repeated triggers and/or other unknown factors, transient systemic autoimmune may become persistent (29–33). The generation of autoantibodies is a key event in the progression to autoimmune and rheumatic bone diseases. Under the influence of T cells or innate triggers, self-tolerance is the first to be interrupted, and the B cell response leads to systemic autoimmunity, producing autoantibodies with pathogenic effects. During this process, T cells activate and differentiate into Th1, Th17 or Tfh cells, release lymphokines, activate macrophages and assist B cells to activate and secrete autoantibodies, which is the main immune abnormality in autoimmune diseases (34). For example, in the pathological process of RA patients, there are high levels of RF and ACPA in the body at this time, and under their pathogenic effects, it gradually progresses to arthritis. As a new type of immunomodulator, iguratimod not only acts on T lymphocytes and B lymphocytes, but also acts on DC dendritic cells, monocytes/macrophages, fibroblasts, and synoviocytes. It also exerts anti-inflammatory effects by inhibiting typical inflammation-related signaling pathways, such as nuclear factor-κB (NF-κB) and IL-17R pathways. Iguratimod can down-regulate the pro-inflammatory factors and some inflammatory factors levels in the circulation, thereby reducing systemic inflammation and improving the overall state of patients (35). Its mechanism of action is consistent with the pathogenesis of autoimmune diseases and rheumatic bone diseases. For example, in the regulation of T cells, continuous use of iguratimod can significantly reduce the number of Th1 cells and Th17 cells in blood circulation, and tend to reduce the number of Tfh cells, while increasing the number of Treg cells. In RA patients, Iguratimod may play an immunomodulatory role by inhibiting inflammatory T cells and promoting the immune response of anti-inflammatory T cells, so as to treat RA (35). Mechanism studies have shown that iguratimod not only inhibits the function of CD4+ T cells in RA patients, but also inhibits the function of CD4+ T cells in RA patients by regulating glucose metabolism, and inhibits the function of CD4+ T cells in RA patients by regulating the Hif1α-HK2 axis (36).

In the regulation of B cells, Iguratimod inhibits the terminal differentiation of B cells by down-regulating protein kinase C (PKC)/early growth response factor 1 (EGR1) (34). Iguratimod can reduce Ig production and inhibit B cell proliferation in mouse spleen B cells, and inhibit B cells and immunoglobulins in SCID mice (37). Iguratimod inhibited IL-4-induced IgM production and IgG1 transformation through its direct effect on B cells. In addition, IgM and IgG produced by B cells were also inhibited in a dose-dependent manner. In SCID HuRAg mice, human IgG was detected in high concentrations of serum, and iguratimod treatment significantly inhibited the accumulation of IgG in mouse serum. Ji et al. found that iguratimod combined with hydroxychloroquine was effective in the treatment of patients with Sjogren ‘s syndrome, which could inhibit B cell activity, prevent immunoglobulin accumulation and improve symptoms (38). Yan et al. found that iguratimod has a variety of regulatory effects on peripheral lymphocytes in MRL/lpr mice. The proportion of peripheral abnormal B220 + T cells and plasma cells decreased after iguratimod treatment, while the proportion of peripheral total B cells recovered and tended to the level of normal BALB/c mice. At the same time, the infiltration of CD20 + B cells in renal tissue as a target organ was significantly reduced after iguratimod treatment. The number of spleen plasma cells in the iguratimod group was significantly lower than that in the placebo group, which echoed the results of serum immunoglobulin detection. The proportion of abnormally elevated germinal center, marginal zone B cells and abnormally reduced follicular B cell subsets in the spleen of mice was partially corrected after iguratimod treatment. The proportion of abnormally increased immature excessive B cells in the spleen of the iguratimod group was also significantly lower than that of the placebo group (39). In Pre-RA with synovial inflammation, iguratimod has a dose-dependent and potential inhibitory effect on synovial arthritis inflammation, mainly targeting IL-17 signal. Iguratimod significantly inhibited the expression of various pro-inflammatory factors triggered by IL-17 in cultured fibroblast-like synovial cells. The inhibition of IL-17 signaling by iguratimod was further related to the decrease of mRNA stability of related genes and the decrease of phosphorylation of mitogen-activated protein kinase (MAPK). Iguratimod mainly targets Act1 and disrupts the interaction between Act1, tumor necrosis factor receptor-associated factor 5 (TRAF5) and IKKi in the IL-17 pathway of synoviocytes (40). In the regulation of dendritic cells, a study reported the effect of iguratimod on the differentiation and maturation of human dendritic cells. Hao et al. found that iguratimod could change the morphology of human monocyte-derived dendritic cells in a dose-dependent manner. When the concentration of Iguratimod was 200ug/ml and 250ug/ml, the time of action on dendritic cells was ≥ 3h, and the expression of CD86 on dendritic cells was significantly decreased. Dendritic cells were co-cultured with allogeneic lymphocytes, and the proliferation ability of lymphocytes was related to the concentration of iguratimod. Iguratimod can inhibit the secretion of IL-12 by dendritic cells in a dose-dependent manner (41). In the regulation of monocyte/macrophage system, studies have shown that iguratimod dereased the ratio of F4/80 + CD86 + and MHCII + on the surface of M1 macrophages, decreased the gene expression level and protein expression level of MCP-1, CD86 and iNOS, decreased the gene expression of IL-1β, IL-6 and TNF-α and decreased the content of IL-1β, IL-6 and TNF-α (42). Further studies showed that Iguratimod significantly inhibited LPS-induced up-regulation of TNFα mRNA and protein levels in NR8383 cells, and also inhibited the transcriptional activity of NF-κB. It is suggested that Iguratimod may reduce the production of TNFα by inhibiting the NF-κB activity of NR8383 induced by LPS (43, 44). Gan et al. found that Iguratimod inhibited Rank1-induced osteoclast differentiation and migration in RAW264.7 cells through NF-κB and MAPK pathways (45).

In the regulation of fibroblasts, Li et al. found that the effect of iguratimod on the secretion of collagen in mouse fibroblast 3T6 cells was time and drug concentration dependent. High concentration could promote the secretion of collagen, and low concentration drug 41.85 mg/L group could reduce the secretion of collagen. Cardiac fibroblasts (CFs) are considered to be semi-professional inflammatory cells that play an immunomodulatory role in the heart (46). Recent studies have shown that Iguratimod inhibits pyroptosis-induced inflammatory response in cardiac fibroblasts through COX2/NLRP3 signaling pathway and alleviates myocardial ischemia/reperfusion injury IM (47).

### 2.2 Anti-inflammatory effect

Iguratimod is anti-inflammatory. Iguratimod inhibited the release of bradykinin in a kaolin-induced arthritis mouse model, as Iguratimod was originally developed as a novel NSAIDs (48). Tanaka et al. reported the anti-inflammatory, analgesic and antipyretic effects of Iguratimod in different animal models (49), the mechanism is related to the inhibition of the metabolism of arachidonic acid metabolite prostaglandin E2 (49), and the inhibition of bradykinin release (50), interleukin (IL)-1 and IL-6 production (50, 51) and selective inhibition of cyclooxygenase-2 activity (52). In several autoimmune disease models, Iguratimod exhibited significant inhibitory effects in experimental autoimmune encephalitis, chronic contractile injury with neuropathic pain, and dextran sodium sulfate-induced colitis (37, 52–59). It is especially important that iguratimod can also selectively inhibit COX-2, thereby reducing the level of prostaglandins and achieving the purpose of anti-inflammation. Of note, iguratimod is less likely to cause gastrointestinal ulcers due to its selective inhibition of COX-2, rather than COX-1 like NSAIDs (52). Iguratimod also inhibits TNF-α-induced inflammatory cytokine secretion and NF-κB activation in human synovial cells, which can interfere with the transfer of NF-κB P65 into the nucleus. By inhibiting NF-κB activity, it affects transcriptional regulation to inhibit the production of cytokines and chemokines, and exert inflammatory inhibition (56, 57). Iguratimod has been found to interfere with TNF-α-induced NF-κB translocation from the cytoplasm to the nucleus, and can inhibit TNF-α-induced IL-6, IL-8 and monocyte chemoattractant protein 1 production. Therefore, iguratimod can affect the expression of inflammatory factors by regulating the NF-κB signaling pathway (58). In regulating neutrophils, Li et al. found that Iguratimod inhibited the expression of CPs by down-regulating PAD in neutrophils of RA patients, and the effect was comparable to MTX and DXM at appropriate concentrations. These findings may provide guidance for more appropriate treatment of RA. Iguratimod inhibits citrulline protein expression in neutrophils of patients with rheumatoid arthritis at an appropriate concentration, similar to MTX and dexamethasone; the inhibitory effect is mediated by the down-regulation of peptidylarginine deiminase, which provides insights into the mechanism of Iguratimod in the treatment of rheumatoid arthritis. This study can guide the treatment of rheumatoid arthritis and promote the identification of other therapeutic targets (59).

### 2.3 Inhibition of immunoglobulin synthesis

Iguratimod inhibits the production of immunoglobulin (IG). Experimental studies on mouse B cells have found that iguratimod can significantly reduce its secreted IgM and inhibit the conversion of IgM to subtype IgG1 induced by lipopolysaccharide (LPS) or IL-4 (37). In human plasmacytoma cell lines, iguratimod can also inhibit IgG production without affecting cell proliferation. In addition, when human peripheral blood B cells are stimulated by autologous T cells and anti-CD3 antibodies, iguratimod can inhibit the production of IgM and IgG in a concentration-dependent manner (60). It has been found that iguratimod inhibits the terminal differentiation of B cells by inhibiting the PKC pathway and the downstream target protein early growth response factor 1 (EGR1). Iguratimod inhibited the expression of the transcription factor BLIMP1, resulting in blocked plasma cell differentiation (34). Iguratimod also inhibits the terminal differentiation of B cells to reduce the production of IG, which is related to improving disease activity and does not affect the proliferation of B cells.

### 2.4 Inhibits cytokine synthesis

Iguratimod can inhibit the production of cytokines. A number of experimental results have confirmed that iguratimod can inhibit a variety of cytokines secreted by a variety of inflammatory cells (61). For example, iguratimod can inhibit the production of TNF-α, IL-6, IL-8 and IL-1β in human peripheral blood monocytes induced by LPS (57). Iguratimod can also interfere with the translocation of NF-κB from the cytoplasm to the nucleus induced by TNF-α, and inhibit the production of TNF-α, IL-6, IL-8, monocyte chemoattractant protein and colony stimulating factor by RA-FLS stimulated by TNF-α or LPS (56). In addition, iguratimod can also inhibit the IL-17 signaling pathway in RA-FLS, which is manifested in the inhibition of the release of various inflammatory cytokines triggered by IL-17 in RA-FLS, such as CCL2, CXCL1, CXCL2, IL-6 and TNF. Studies have found that iguratimod has a significant inhibitory effect on synovial inflammation of collagen-induced arthritis in a dose-dependent manner.

Iguratimod has a specific inhibitory effect on IL-17. It inhibits IL-17-mediated signal transduction by destroying the interaction between Act1, a key adaptor protein of IL-17 signal transduction, and tumor necrosis factor receptor-associated factor 5, thereby inhibiting IL-17-induced expression of various inflammatory factors. Act1 (NF-κB activator 1) is a very important adaptor protein and plays an important role in the development of arthritis (40). In addition, macrophage migration inhibitory factor (MIF) can stimulate the production of IL-17 by regulating IL-17 inducers and promote Th17 cell activity that mediates autoimmune (62). MIF is a pleiotropic cytokine involved in a variety of inflammatory and neoplastic diseases. Studies have shown that MIF plays an important pathological role in inflammatory diseases such as rheumatoid arthritis and systemic lupus erythematosus (63, 64). Iguratimod can interact with MIF trimer, inhibit MIF tautomerase activity, and inhibit MIF-induced proinflammatory responses, including B cell proliferation, monocyte factor release, and inflammation relief. Therefore, iguratimod has been identified as an anti-inflammatory MIF inhibitor (65). In summary, iguratimod inhibits inflammatory cytokines through a variety of ways.

### 2.5 Bone protection

Iguratimod has bone protection, which is manifested as inhibiting bone resorption and promoting bone formation. Studies have shown that iguratimod increases the expression of osteogenic transcription factors (osterix, OSX) and enhances bone morphogenetic protein 2 (BMP2) -mediated bone formation. OSX plays a crucial role in osteoblast differentiation. BMP2 can activate OSX by inducing the downstream transcription factor Dlx5 (distal-less homeobox 5). Iguratimod can promote osteoblast differentiation by increasing the expression of OSX and Dlx5. The interaction between p38-MAPK pathway and BMP2-Smads pathway promoted the phosphorylation of OSX. P38-MAPK is a member of the MAPK superfamily and participates in the early process of osteoblast proliferation through the phosphorylation of Dlx5, runt-related transcription factor 2 (RUNX2) and OSX. Iguratimod can enhance the activation of p38 and promote osteoblast differentiation (66, 67). It was found that iguratimod significantly inhibited RANKL-induced osteoclast differentiation, migration and bone resorption in RAW264.7 cells in a dose-dependent manner. The mechanism is related to the activation of mitogen-activated protein kinase and NF-κB pathway (45). Iguratimod can also inhibit the expression of MMP-1 and MMP-3 in RA-FLS and inhibit the invasiveness of RA-FLS, thereby reducing bone destruction (68). In addition, iguratimod significantly down-regulated the mRNA of osteoclast-related genes, such as tartrate resistant acid phosphatase (TRAP), cathepsin K and calcitonin receptors, and inhibited the expression of chemokines to inhibit bone resorption (69).

In regulating chondrocytes, Peng et al. based on the Wnt/β-catenin signaling pathway, explored the effect and mechanism of iguratimod on interleukin (IL) -1β-induced rat osteoarthritis cartilage matrix metabolism at the cellular and molecular levels. They found that Wnt/β-catenin signaling pathway was overexpressed in IL-1β-induced rat degenerative chondrocytes, which promoted the degradation and metabolism of chondrocyte matrix and inhibited its synthesis function. Iguratimod protects IL-1β-induced rat degenerative chondrocytes by regulating the Wnt/β-catenin signaling pathway (70).

In regulating osteoclasts and osteoblasts, studies have shown that iguratimod can effectively reduce bone loss in ovariectomized mice; iguratimod inhibits RANKL-induced PPAR-γ/c-Fos pathway activation in mouse bone marrow mononuclear macrophages, which in turn affects the expression of key transcription factors and functional proteins in downstream osteoclasts, and ultimately affects the formation and function of osteoclasts. Iguratimod can affect the differentiation of mesenchymal stem cells into adipocytes by inhibiting the expression of PPAR-γ (71).

### 2.6 Anti-pulmonary fibrosis

Pulmonary fibrosis is a common lung disease in rheumatism. Pulmonary fibrosis is caused by a strong inflammatory response after lung injury, which is characterized by the destruction of alveolar structure and abnormal deposition of extracellular matrix. In the early stage of the development of alveolar inflammation to pulmonary fibrosis, pro-inflammatory cytokines such as TNF-α, IL-1 and IL-6 increase, which play an important pathogenic role, and inflammatory cells also induce the production of matrix metalloproteinases (72). Iguratimod can inhibit the expression of TNF-α, IL-1, IL-6 and MMP-9 to reduce bleomycin-induced alveolar inflammation and pulmonary fibrosis in mice, suggesting that iguratimod may become an effective strategy for the treatment of pulmonary fibrosis (73).

**Figure 2:**
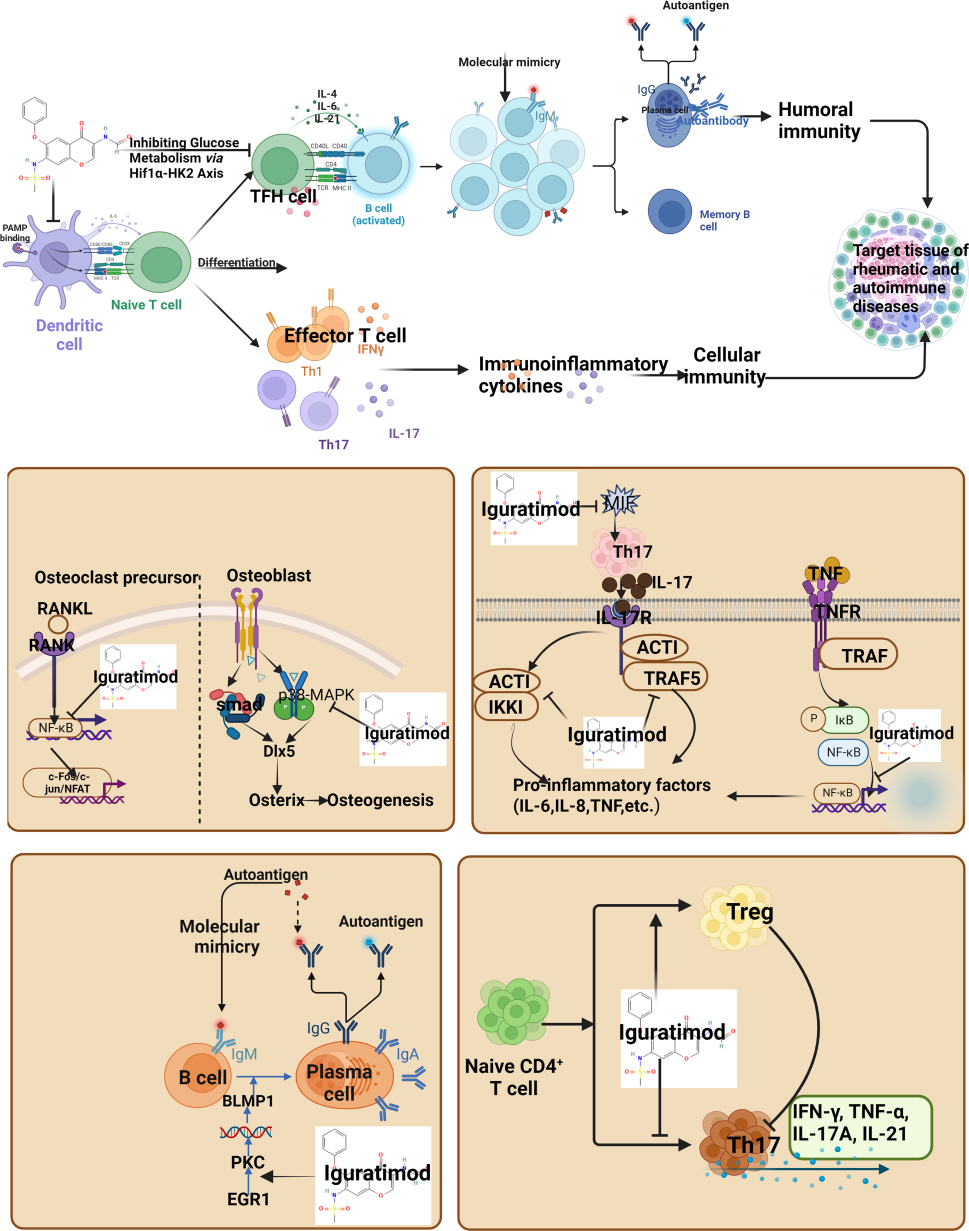
Regulatory Mechanism of Iguratimod on Signaling Pathway (IFN, interferon; TNF, Tumor necrosis factor; IL, Interleukin; Th, helper T cells; Treg, Regulatory T cells).

The mechanism of iguratimod on signaling pathway were summarized in **Figure 2**.

## 3 Iguratimod in the treatment of rheumatic bone diseases and autoimmune diseases

### 3.1 Iguratimod in the treatment of RA

Rheumatoid arthritis (RA) is a chronic and systemic autoimmune disease. The main clinical features of the disease are erosive and symmetrical polyarthritis, in which the involvement of hands, wrists, knees, ankles and feet is the most common (17). The basic pathological changes of RA include chronic synovitis, pannus, articular cartilage and bone destruction, which eventually lead to joint deformity and loss of function (74–76). The etiology of RA is very complex, mainly including environmental factors, genetic factors, environment-genetic interaction, and epigenetic modification, etc. These factors and their interactions jointly lead to the pathogenesis of RA (77). The pathogenesis of RA can be divided into three stages—firstly, environmental factors such as smoking or infection act on individuals with specific genetic backgrounds, destroying the immune tolerance of the body, autoimmune reactions occur in the body, and RF and ACPA can appear in the serum and other autoantibodies (78). Then, the inflammation in the joint synovium continued to increase, many pannus formed in the joint cavity, the synoviocytes proliferated, and gradually developed into chronic inflammation; finally, the joint bone was destroyed and remodeled, resulting in joint deformity, and the patient’s prognosis was poor (79). Studies have shown that the main pathological mechanisms of RA include immune response, inflammatory factors, cartilage and bone destruction (62, 80).

Current studies have shown that iguratimod can selectively inhibit COX-2 and nuclear transcription factor-κB (NF-κB) to reduce the inflammatory response and is used in primary or secondary drug resistance of RA (81). In addition, synovitis is the characteristic pathological manifestation of RA, and the abnormal proliferation of fibroblast-like synoviocytes (FLS) is an important factor in the occurrence and development of RA synovitis (64). Recent studies have shown that iguratimod can also effectively inhibit the proliferation of RA-FLS (82). Wang et al. have proved that iguratimod can selectively inhibit the expression of COX-2 mRNA and c-fos mRNA, thereby inhibiting the proliferation of RA-FLS, and the inhibitory effect is in line with the dose-effect relationship (83). DU et al. found that iguratimod reduced the expression of matrix metalloproteinase-1 (MMP-1) and MMP-3, and inhibited the excessive proliferation of FLS (68). Meng et al. clinical studies have shown that iguratimod can reduce the release of vascular endothelial growth factor and increase the production of endostatin to reduce synovial angiogenesis (84). LIN et al. found that iguratimod can significantly inhibit the invasion behavior of RA-FLS and promote its apoptosis through the mitogen-activated protein kinase (MAPK) signaling pathway through cell metastasis experiments. In addition, iguratimod also inhibits the occurrence and development of synovitis by inhibiting inflammation-related cytokines (85).

Clinical studies have shown that iguratimod can reduce the expression of inflammatory factors such as IL-9 and IL-8 and reduce the level of RANKL by reducing serum interleukin (IL) -17. At the same time, iguratimod can directly act on RANKL and coordinate with IL-17 to regulate the OPG/RANKL/RANK axis system and delay bone destruction. The combination of MTX can significantly increase the level of OPG, the effect is better (40). Feng et al. (86) have shown that iguratimod can inhibit the expression of RANK, tartrate-resistant acid phosphatase, cathepsin K, activating protease-1 and other genes that play an important role in osteoclast differentiation and activation, thereby inhibiting the proliferation and differentiation of osteoclasts. The positive anti-citrulline protein antibody is also closely related to RA bone loss. Iguratimod can dose-dependently down-regulate peptidylarginine deiminase 2 (PADI2) and PADI4 in neutrophils, thereby inhibiting the expression of citrullinated proteins and reducing bone loss (59).

Methotrexate is an anchoring drug for the treatment of RA, and MTX is often combined with iguratimod in clinical practice. SHRESTHA et al. (87) meta-analysis showed that iguratimod was similar to MTX in terms of therapeutic effect, disease status and adverse reactions at 24 weeks, and iguratimod may be a potential alternative to MTX. In terms of clinical efficacy and safety, a number of meta-analyses explored the efficacy and safety of iguratimod monotherapy in the treatment of RA. For example, the latest study by Ouyang et al. included 4302 RA patients from 38 RCTs (88). It was found that compared with the MTX subgroup alone, although the evidence of DAS28-ESR results was insufficient, the use of Iguratimod alone had obvious advantages in improving ACR20 and DAS28-ESR. Compared with standard therapy, Iguratimod + MTX showed significant benefits in improving ACR20, ACR50, ACR70 and DAS28-ESR scores (89). When the intervention (Iguratimod alone or Iguratimod + MTX) continued for 52 weeks, it showed excellent efficacy in improving ACR20 in patients without significant adverse events. It is worth noting that Iguratimod or Iguratimod + MTX has obvious advantages in improving ACR20 of first-diagnosed RA, and Iguratimod + MTX has obvious advantages in improving DAS28-ESR of refractory RA. In addition, Iguratimod + MTX does not increase the risk of leukopenia, but can reduce the risk of liver function tests (LFT), regardless of the age or stage of RA (89). In terms of iguratimod combined with MTX, Zeng et al. included 31 randomized controlled trials involving 2776 patients. Compared with MTX alone, Iguratimod + MTX has higher ACR20, ACR50 and ACR70, and lower DAS28. Compared with conventional treatment, Iguratimod + MTX may be a safer and more effective treatment for RA patients. When the intervention method is (Iguratimod 25 mg Bid, MTX 10-25 mg once a week), and the intervention lasts for at least 12 weeks, the efficacy can be achieved without significant adverse events (90). A recent largest sample authenticity study was conducted from March 2012 to August 2015.A total of 1759 patients with active RA were recruited from 48 hospitals or clinical research centers in China as subjects. This is a relatively rare large sample study in the field of RA at home and abroad. In this study, the ACR 20 response rate at week 24 was the primary endpoint, and the ACR 50 and ACR 70 response rates and the changes of DAS28 and HAQ at week 24 were the secondary endpoints. The results showed that the ACR response rate gradually increased with the treatment time. At week 12, the response rates of ACR 20, ACR 50 and ACR 70 were 62.2%, 29.5% and 11.0%, respectively. At week 24, the response rates were 71.9%, 47.4% and 24.0%, respectively. In addition, compared with baseline, the DAS28 score and HAQ score of the subjects decreased significantly at week 12 and week 24 (P < 0.001), suggesting that iguratimod could significantly improve the clinical symptoms and physical function of RA patients (91).

In addition, the current study also found that iguratimod has good clinical efficacy and safety for RA with interstitial lung disease. Lung is the most common extra-articular organ involved in RA, and its lesions are mainly interstitial lung disease. Long-term low-dose application of MTX also has adverse reactions to interstitial lung disease (92). Han et al. (93) found through animal experiments that iguratimod can improve bleomycin-induced idiopathic pulmonary fibrosis in mice by inhibiting inflammatory response. Hao et al. (94) short-term clinical observation showed that iguratimod had a definite effect on RA complicated with chronic interstitial pneumonia, and the incidence of adverse reactions was low, without infection. Ma et al. (95) used tripterygium glycosides combined with iguratimod to treat RA with interstitial lung disease, which can effectively reduce the expression of peripheral blood serum hypoxia-inducible factor and IL-22, and has obvious advantages over the simple use of tripterygium glycosides. Zhao et al. (73) have shown that iguratimod can improve lung fibrosis by inhibiting the expression of MMP-9, IL-1, IL-6 and other factors. Therefore, when patients have pulmonary lesions, iguratimod can be given priority. RA has a certain impact on the cardiovascular system, that is, the cardiac conduction system. An important factor in cardiac dysfunction in RA patients is the imbalance between oxidation and antioxidant systems. A clinical study of refractory RA has shown that the combination of iguratimod and MTX can increase superoxide dismutase, reduce total antioxidant capacity (96), and inhibit cardiovascular disease complicated by RA in controlling oxidative stress. Randomized controlled trials (RCTs) of iguratimod in RA are presented in **Table S1** (see **Supplementary Material**).

### 3.2 Iguratimod in the treatment of pSS

Primary Sjögren ‘s syndrome (pSS) is a chronic inflammatory autoimmune disease characterized by progressive exocrine gland damage and lymphocyte proliferation, and can involve multiple organs and systems (97). Primary Sjgren ‘s syndrome is a global disease with a prevalence of 0.33% ~ 0.77%. The age of onset is mostly 40 ~ 50 years old, and the ratio of male to female is 1:9 ~ 1:20. It is the most common autoimmune connective tissue disease in middle-aged and elderly people (98). The prognosis of patients with lesions confined to the salivary gland, lacrimal gland, skin and mucosal exocrine glands is better, and the prognosis of patients with pulmonary interstitial fibrosis, central nervous system lesions, renal insufficiency and lymphoma is poor (99). There is no satisfactory treatment for primary Sjogren ‘s syndrome. Whether it is dry, fatigue or systemic damage, there is a lack of effective drugs demonstrated by evidence-based medicine. The drugs currently used are mostly empirical therapeutic drugs (100, 101). Among them, hydroxychloroquine has certain retinal toxicity, and there are many adverse reactions of glucocorticoids, which affect its long-term application (102).

It was found that Iguratimod could reduce the degree of lymphocyte infiltration in exocrine glands and significantly reduce the level of serum IL-17 in NOD mice, and the effect was more obvious with the increase of dose. It is suggested that Iguratimod has a protective effect on exocrine gland tissue in model mice, and can improve exocrine gland function and improve its quality of life (103). Qi et al. found that Iguratimod can effectively improve the dry symptoms of NOD mice and reduce the pathological damage of submandibular gland (104); they speculated that Iguratimod may inhibit the inflammatory response of submandibular gland in Sjogren ‘s syndrome mice by inhibiting the activation of NF-κB signaling pathway. In the regulation of B cells, Jiang et al. detected the expression of B cell surface molecules in patients with Sjogren ‘s syndrome treated with Iguratimod or conventional therapy alone, and believed that Iguratimod treatment of Sjogren ‘s syndrome may inhibit the secretion function of BAFF-R positive B cells and type 2 memory B cells in the activated phase, and reduce the pathological infiltration of B cells in the ectopic germinal center and blood (105). It has been reported that the number and percentage of peripheral blood memory B cells (CD + 19CD + 27) and their mediated immunoglobulin expression in pSS patients are higher than those in normal people (88). In terms of anti-drying combined with pulmonary fibrosis, Han et al. (106) found that Iguratimod improved the degree of fibrosis in bleomycin-induced pulmonary fibrosis model mice by inhibiting inflammatory response in lung tissue. Zhao et al. (73) found that Iguratimod may improve the process of lung inflammation and fibrosis by inhibiting the expression of TNF-α, IL-1, IL-6 and MMP-9 in bleomycin-induced pulmonary fibrosis mice. The results of cell and animal experiments of Zhu et al. showed that Iguratimod can inhibit TGF-β1-mediated human lung fibroblast activation and collagen secretion through the Smad3/p300 pathway, and can be used as an effective anti-fibrotic drug to delay the progression of pulmonary fibrosis (107).

In the clinical application of Iguratimod in primary Sjogren ‘s syndrome, at present, clinical studies have found that Iguratimod alone or combined with one or two drugs in methylprednisolone, hydroxychloroquine sulfate and total glucosides of paeony in patients with Sjogren ‘s syndrome has good efficacy and safety. In the efficacy and safety evaluation trial of primary Sjogren ‘s syndrome, the observation group received methylprednisolone (8 mg, qd) + Iguratimod (25 mg, qd), and the control group received methylprednisolone (8 mg, qd) + HCQ (0.2 g, bid) for 3 months. The total effective rate of the observation group was 93.6%, higher than 76.6% of the control group. The ESR, IgG, RF, ESSDAI and ESSPRI scores of the observation group were lower than those of the control group. Platelet count, Schirmer test and saliva flow rate were higher than the control group (108). The results showed that Iguratimod was more effective than HCQ in the treatment of pSS, and Iguratimod could effectively reduce serum B lymphocyte activity (88). Liang et al. (109) included 12 randomized controlled trials, including 1004 patients. The results showed that the efficacy of Iguratimod combined with methylprednisolone in the treatment of primary Sjogren ‘s syndrome was better than that of hydroxychloroquine sulfate combined with methylprednisolone in improving the total effective rate of treatment, reducing ESSDAI score, reducing ESSPRI score, increasing Schirmeri test value, increasing salivary flow rate, reducing IgG, reducing ESR, reducing RF, increasing PLT, reducing CD + 19 CD + 27 B cell percentage, and reducing anticardiolipin antibody. Iguratimod can be combined with other antirheumatic drugs such as methylprednisolone, hydroxychloroquine sulfate and total glucosides of paeony to treat Sjogren ‘s syndrome. Compared with the control group treated with hydroxychloroquine sulfate monotherapy, the levels of BAFF-R and CD-38 IgD in the observation group were lower than those in the control group after the addition of Iguratimod. It is believed that Iguratimod can reduce B cell activity, thereby reducing immunoglobulin secretion and improving symptoms (38). In the case of methylprednisolone and hydroxychloroquine sulfate, combined with Iguratimod can significantly reduce ESSPRI score, ESSDAI score, Ig, RF and ESR, increase platelet level and do not increase the incidence of adverse reactions. In the study of senile PSS. Iguratimod combined with hydroxychloroquine and total glucosides of paeony can effectively improve the exocrine gland function, improve the symptoms of dry mouth and dry eyes, reduce the levels of B lymphocytes, immunoglobulin, RF and inflammation in elderly patients with primary Sjogren ‘s syndrome, and it is safe and reliable (110, 111). In addition, interstitial pneumonia and pulmonary fibrosis are common lung lesions in Sjogren ‘s syndrome. For patients with interstitial pneumonia secondary to Sj gren ‘s syndrome, the addition of Iguratimod on the basis of symptomatic treatment can improve the lung function of patients with Sj gren ‘s syndrome and interstitial lung disease [increase forced vital capacity (FVC) and maximum mid-expiratory flow rate (MMF)], control inflammatory reactions and have mild adverse reactions (112, 113). A meta-analysis of 2258 participants included in 19 randomized controlled trials (RCTs) showed. Iguratimod could effectively reduce ESSPRI score [WMD − 1.93 (95% CI: − 2.33, − 1.52), P < 0.00001], ESSDAI score [− 1.39 (− 1.81, − 0.98), P < 0.00001], Schirmer test [− 1.77 (0.85, 2.70), P = 0.0002], RF [− 5.78 (− 7.59, − 3.97), P < 0.00001], ESR level [− 7.05 (− 9.84, − 4.26), P < 0.00001]. And will not increase the occurrence of adverse events (114). Another meta-analysis showed that Iguratimod improved the clinical symptoms of pSS patients, including inflammatory indicators (ESR, IgG and RF levels), PLT count, salivary gland and lacrimal gland secretion function (salivary flow rate and Schirmer ‘s test results) and disease index (ESSDAI and ESSPRI), when administered in conjunction with HCQ + GC treatment, does not increase the risk of AE. Considering the limitations of existing trials, more long-term, multi-center and high-quality randomized controlled trials are needed to evaluate the efficacy and safety of Iguratimod in patients with pSS (115).

In summary, we can see that a number of clinical studies have applied iguratimod alone or in combination with one or two of methylprednisolone, hydroxychloroquine sulfate, and total glucosides of paeony to patients with Sjogren ‘s syndrome, which can improve the total effective rate of treatment, improve dry symptoms, reduce globulin, reduce inflammation and antibody levels, increase platelets, improve lung function, and have low incidence of adverse reactions. Iguratimod can treat Sjogren ‘s syndrome by reducing glandular inflammation and pathological damage, inhibiting B lymphocytes and anti-pulmonary fibrosis. Randomized controlled trials (RCTs) of iguratimod in pSS are presented in **Table S2** (see **Supplementary Material**).

### 3.3 Iguratimod in the treatment of SLE and lupus nephritis

Systemic lupus erythematosus (SLE) is an autoimmune-mediated systemic inflammatory disease (116). Lupus nephritis (LN) is one of the most common complications of SLE and a major predictor of poor prognosis (117). Therefore, in-depth research on the pathogenesis of lupus nephritis will bring hope for future precise treatment that directly targets specific cells, autoantibodies, cytokines and chemokines to regulate inflammation and tissue damage (118). As an autoimmune disease involving multiple organs and systems, the abnormal activation of T and B lymphocytes in SLE patients causes inflammatory injury (119), and iguratimod has the effect of regulating T, B lymphocytes and antigen presenting cells, so iguratimod has potential for SLE treatment.

Studies have shown the preventive and protective effects of iguratimod on systemic lupus erythematosus (SLE) model mice. For example, the levels of serum antinuclear antibody, anti-ds-DNA antibody, anti-RNP/sm antibody, urine protein positive rate, serum urea nitrogen and serum creatinine in the iguratimod intervention group were significantly lower than those in the model control group, and the renal pathological changes in the iguratimod intervention group were not obvious. Iguratimod can inhibit the production of serum antibodies, improve the urine protein, serum urea and serum creatinine levels in SLE model mice, and has preventive and protective effects on SLE model mice (120). In terms of the mechanism of treatment of SLE, the study showed that Iguratimod had an intervention effect on cGVHD lupus nephritis mice. The results showed that compared with the cGVHD untreated group, the body weight of the mice in the Iguratimod group gradually increased, and the urine protein gradually decreased. After the experiment, serum ANA, anti-dsDNA antibody and IgG immune complex deposition in the kidney decreased, and renal pathology was improved. At mRNA level, TLR9 expression was decreased (121). At the mRNA and protein levels, NF-κB expression decreased, activity decreased, and downstream inflammatory factors were down-regulated, while Nrf2 expression increased and downstream antioxidant factors were up-regulated. Yan et al. found that iguratimod can significantly alleviate lupus-like disease in MRL/lpr mice. The time and level of proteinuria in mice after treatment with iguratimod were significantly lower than those in the placebo group (122). The above results also confirmed the inhibitory effect of iguratimod on humoral immunity in mice. The proportion of peripheral abnormal B220 + T cells and plasma cells decreased after iguratimod treatment, while the proportion of peripheral total B cells recovered and tended to the level of normal BALB/c mice. Meanwhile, the infiltration of CD20 + B cells in renal tissue as a target organ was significantly reduced after iguratimod treatment. The proportion of abnormally elevated germinal center, marginal zone B cells and abnormally reduced follicular B cell subsets in the spleen of mice was partially corrected after iguratimod treatment. The proportion of abnormally increased immature excessive B cells in the spleen of the iguratimod group was also significantly lower than that of the placebo group. The above effects of cyclophosphamide were not obvious. The levels of BAFF, IL-6, IL-21 and IL-17 A in spleen cells and serum of MRL/lpr mice were significantly decreased after iguratimod administration, which was similar to that of cyclophosphamide. No obvious side effects of iguratimod on MRL/lpr mice were observed during long-term administration (20 weeks). Iguratimod can alleviate lupus-like disease in MRL/lpr mice, and its mechanism may play a role in correcting the abnormal B cell differentiation function of MRL/lpr mice by non-anti-proliferation. The results of this study suggest that iguratimod may be a potential new drug for lupus treatment.

Based on network pharmacology and molecular docking, some studies have revealed the potential therapeutic mechanism of Iguratimod on SLE protein targets through PI3K-AKT signaling pathway, MAPK signaling pathway and FoxO signaling pathway (123). In the study of lupus kidney, Chen et al. used Pristane to induce the kidney of systemic lupus erythematosus (SLE) mice. Iguratimod can significantly protect the kidney tissue of SLE mice and alleviate the condition of SLE mice. In terms of immune cell regulation, iguratimod reduced the severity of nephritis in PI mice in a dose-dependent manner. Proteinuria continued to decrease, the pathology of glomerulonephritis and tubular nephritis was significantly reduced, and glomerular immune complex deposition was reduced (124). In addition, Iguratimod reduced serum anti-dsDNA and total IgG and IgM levels in mice. It is worth mentioning that the efficacy of 30mg/kg/d of Iguratimod is equivalent to or even better than 100mg/d of mycophenolate mofetil. In addition, Iguratimod can decrease the percentage of Th17 cells and increase the percentage of Treg cells (125). Studies have shown that Iguratimod reduced immune deposition along the basement membrane of renal tubules, inhibited tubulointerstitial infiltration of inflammatory cells, and alleviated renal tubular injury in MRL/lpr mice. Iguratimod inhibited the expression of FSP-1 and increased the expression of E-cadherin in renal tubular epithelial cells. In HK2 cells cultured with TGF-β1, iguratimod treatment not only reversed cell morphological changes, but also prevented down-regulation of E-cadherin and up-regulation of fibronectin. In addition, Iguratimod inhibited the phosphorylation of TGFβRII, Smad2/3 and p38 MAPK in TGF-β1-treated HK2 cells and blocked the nuclear translocation of β-catenin. Iguratimod alleviates tubulointerstitial lesions in LN patients, especially tubulointerstitial fibrosis, and may have the potential to inhibit the progression of tubulointerstitial fibrosis in LN (39).

On the clinical side, one study recruited eligible patients with refractory LN – which were defined as failure or relapse on at least two immunosuppressant treatments. After enrollment, they replaced their previous immunosuppressant with iguramod (25 mg twice daily) without increasing the dose of steroids. The renal response rate at week 24 was 92.3% (12/13), of which 38.5% (5/13) achieved CR and 53.8% (7/13) achieved PR. We then continued to follow patients who responded for up to 144 weeks. Renal recurrence occurred after initial PR in 25% of patients (3/12). Estimated glomerular filtration rates remained stable during follow-up in all patients. One patient had a serious adverse reaction (anemia) but made a full recovery after discontinuing iguratimod. This study supports the potential of iguramod in the treatment of refractory LN. Iguratimod may be a promising drug candidate for this condition (126).

At present, studies have shown that after half a year of combined treatment with iguratimod and Kunxian Capsules, urine protein turned negative and blood pressure returned to normal. This case suggests that when the conventional treatment plan is ineffective for lupus nephritis, if the patient gives informed consent, another approach can be found, and Airamod combined with Kunxian Capsules is another alternative treatment plan (127). A comparison of iguratimod, conventional cyclophosphamide and sequential azathioprine in the treatment of active lupus nephritis has also been published recently, and more relevant clinical research reports will give us more evidence in the future (128).

### 3.4 Iguratimod for the treatment of IgG4-related disease

IgG4-RD is a chronic progressive autoimmune disease characterized by elevated serum IgG4 levels and infiltration of IgG4-positive cells in multiple organs at a relatively early stage (129, 130). Current studies have shown that activated B cells and cytotoxic CD4 T cells together lead to tissue fibrosis and functional impairment (131). However, iguratimod can inhibit the terminal differentiation of B cells and reduce the production of autoantibodies. ZHANG et al. (132) found through a prospective clinical study that iguratimod combined with corticosteroids had a significant effect on mild IgG4-RD. LIU et al (133) conducted a more in-depth retrospective clinical study that iguratimod can improve the clinical symptoms and serum levels of IgG and IgG4 in patients with relapsed or refractory IgG4-RD, and reduce the volume of involved glands, It is also more reliable in terms of security. The pancreas is the organ most commonly involved in IgG4-RD. Experimental studies by HOU et al. (134) showed that iguratimod can inhibit the Nod-like receptor protein 3 (NLRP3) inflammasome, and play a protective role in the severe acute pancreatitis mouse model by inhibiting the NF-κB signaling pathway (135). Liu et al. 17 cases of IgG4- A retrospective single-center study of RD patients found that serum IgG4 decreased significantly from 708 (321-902) mg/dl at baseline to 446 (138-396) mg/dl at 24 weeks (P = 0.0016). IgG4-RD RI decreased significantly from 9.79 ± 3.07 at baseline to 3.57 ± 1.09 at 24 weeks (P < 0.0001). Overall, 2 (14.3%) patients achieved CR, 11 (78.6%) patients achieved PR, and 1 (7.14%) patient did not respond to treatment at week 24. Serum IgG levels and salivary gland major diameters also decreased significantly at 12 and 24 weeks after treatment (133). Our previous work showed that at week 24, 9 (30.0%) patients achieved complete remission, 17 (56.7%) patients achieved partial remission, and 4 (13.3%) patients did not respond to treatment. IgG4-RD RI, serum IgG and IgG4 levels decreased significantly at weeks 12 and 24 after treatment, as well as CD3+ CD8+ T cells, plasmablasts/plasma cells, and memory B cells. LC-MS-based plasma metabolomic profiling revealed significant changes between untreated patients and healthy donors, which closely resembled the normal state after treatment (132).

### 3.5 Iguratimod and ankylosing spondylitis

Ankylosing spondylitis (AS) is a chronic inflammatory disease that invades the spine and joints. As a chronic progressive autoimmune disease characterized by aseptic inflammation of the axial joints, cytokines such as IL-1, IL-6, IL-17, IL-18, TNF-α, and vascular endothelial growth factor are associated with AS activity (136, 137). Studies have shown that iguratimod can inhibit the production of inflammatory factors such as interleukin-1 (IL-1), IL-6, IL-17 and TNF-a, while TNF-a is an important inflammatory factor in the pathogenesis of AS.At the same time, the L-23/IL-17 pathway has been proved to be of great significance in the pathogenesis of AS (59, 138–140), so iguratimod may be a promising drug for the treatment of AS.

Current evidence suggests that Iguratimod can regulate the RANKL/RANK/osteoprotegerin system by reducing RANKL expression (141–143). In addition, Iguratimod could inhibit RANKL-induced osteoclast formation, differentiation, migration, and bone resorption in RAW264.7 cells (45). Furthermore, Iguratimod stimulated osteoblastic differentiation of stromal cell line (ST2) and osteoblastic cell line (MC3T3-E1) in the presence or absence of recombinant human bone morphogenetic protein-2 (rhBMP-2) (144). Oral administration of Iguratimod to mice also promotes rhBMP-2-induced bone formation by increasing the expression of Osterix, an important transcription factor for osteoblast differentiation (144). Taken together, Iguratimod has an anabolic effect on bone metabolism by inhibiting bone fragment formation and promoting osteoblast differentiation. Current evidence suggests that Iguratimod has anabolic effects on bone metabolism.

In a clinical study, a meta-analysis A total of 4 studies were included (n=176). The results showed that iguratimod treated ankylosing spondylitis and improved back pain (WMD=1.80, 95%CI was 1.43-2.17, P<0.000 01), ESR (WMD=14.94, 95%CI was 6.18-23.70, P= 0.000 8), BASDAI (WMD=0.80, 95%CI is 0.49~1.11, P<0.00001), BASFI (WMD=0.72, 95%CI is 0.30~1.15, P=0.0009) slightly better than traditional synthesis Conventional synthetic disease-modifying anti-rheumatic drugs (cs DMARDs), morning stiffness time and cs DMARDs were not significantly different, and the incidence of adverse reactions was lower than cs DMARDs (RR=0.43, 95%CI was 0.26~0.71, P=0.001). Therefore, Iguratimod combined with non-steroidal anti-inflammatory drugs (NSAIDs) in the treatment of ankylosing spondylitis improves clinical evaluation indicators slightly better than cs DMARDs combined with NSAIDs, and the incidence of adverse reactions is lower than that of cs DMARDs. Therefore, although the current evidence from previous studies is not strong enough, more clinical studies with larger sample size, longer duration, and clinical data (including radiology and MRI results) are needed to evaluate the effect of Iguratimod in the treatment of AS (145). A clinical research found that iguratimod may significantly reduce the symptoms and signs of patients with active SpA. It improved physical function and quality of life in these patients, and was overall safe and well tolerated (146).

Studies by Luo et al. and Qiu et al. have shown that Iguratimod can effectively improve the clinical symptoms of refractory AS patients, and the proportion of patients meeting the ASAS 20 remission criteria is increasing (147, 148). In the study by Luo et al., refractory AS patients were defined as those who failed NSAID therapy for 2 weeks and had high disease activity, i.e., ankylosing spondylitis disease activity score (ASDAS) >2.1 or Bass Ankylosing spondylitis disease activity index (BASDAI) >4.0 (147). In the study by Qiu et al., refractory AS patients were defined as patients who failed conventional NSAID and DMARDs treatment for at least 3 months (148). In the study by Huang et al., 34 AS patients with BASDAI ≥ 4 received Iguratimod treatment for 6 months. After 3 and 6 months of treatment, C-reactive protein (CRP), erythrocyte sedimentation rate (ESR) and BASDAI, The Bath Ankylosing Spondylitis Functional Index (BASFI) score decreased significantly (149). Other studies have shown an increase in the proportion of patients meeting ASAS20 criteria after at least 12 weeks of Iguratimod treatment, which was significantly higher than in the conventional DMARD group (149–151). In summary, all studies have shown that Iguratimod has a good short-term effect on AS with few side effects, including mild gastrointestinal side effects, leukocytosis, and elevated transaminases (149–152). It is worth noting that the currently available RCTs are all small-scale trials using traditional DMARDs, whether using SASP alone or in combination with MTX (MTX) as a control drug, and there is no mention of the effect of Iguratimod on bone structure changes in AS. Influence (148–152). Randomized controlled trials (RCTs) of iguratimod in AS are presented in **Table S3** (see **Supplementary Material**).

### 3.6 Iguratimod in the treatment of systemic sclerosis

Systemic sclerosis (SSc), also known as scleroderma, is a systemic autoimmune disease characterized by localized or diffuse skin thickening and fibrosis (153). The lesion is characterized by skin fibrosis and vascular onion-like changes, eventually leading to skin sclerosis and vascular ischemia (154). This disease is clinically characterized by localized or diffuse skin thickening and fibrosis. In addition to skin involvement, it can also affect internal organs (heart, lung and digestive tract). As an autoimmune disease, it is often accompanied by antinuclear antibodies, anticentromere antibodies, anti-Scl-70 and other autoantibodies (155). Recent studies have shown that Iguratimod can improve bleomycin-induced pulmonary fibrosis by partially inhibiting the expression of MMP-9 and inhibiting mouse fibroblast-to-myofibroblast transformation and immunoglobulin secretion (156–158). Other studies have shown that iguratimod can reduce general inflammation and improve lung function in patients with RA-associated interstitial lung disease, 18 suggesting that iguratimod has potential anti-fibrotic effects and may hold promise for the treatment of cutaneous fibrosis in SSc.

Xie et al. found that Iguratimod inhibited TGF-β1-induced cell proliferation and migration in a dose-dependent manner and promote cell apoptosis. Iguratimod partially reversed TGF-β1-induced upregulation of fibrosis markers and phosphorylation of smad2 and smad3, and blocked p-Smad3 nuclear translocation, suggesting that Iguratimod may regulate TGF-β1 β1/smad pathway to inhibit the activation of dermal fibroblasts (159). Iguratimod inhibits dermal fibroblast activation and skin fibrosis at least in part by modulating the TGF-β1/smad pathway in experimental SSc models and may be a promising therapeutic agent for SSc. Bloom et al. found that iguramod synergizes with glucocorticoids to attenuate experimental autoimmune encephalitis, a model of multiple sclerosis (65).

### 3.7 Iguratimod for multiple sclerosis

Multiple sclerosis (MS) is an autoimmune disease of the central nervous system characterized by inflammatory cell infiltration, neuronal degeneration, axonal damage, and reactive gliosis (160). Its cause and mechanism have not been fully ascertained, and it is generally believed to be the result of multiple factors such as genetic susceptibility, environment, virus infection, lifestyle, and immunity (161). The immune mechanism is the final link in the pathogenesis of MS, and it is also the most important link leading to neuronal degeneration and axonal injury. A variety of causes act on genetic susceptibility, causing the activation of the peripheral immune system, and the proliferation of effector T cells under the action of antigen presentation response (162–164). Activated CD4 T cells mediate and amplify the inflammatory response by producing various pro-inflammatory factors. Under the influence of adhesion molecules, integrins, cytokines and chemokines secreted by various immune cells, tight junction proteins on the blood-brain barrier produce gaps and endothelial cell function (165, 166). Alterations occur, leading to increased permeability of the blood-brain barrier and subsequent entry into the central nervous system (167).

Recent studies have shown that oral administration of Iguratimod to mice immunized with myelin oligodendrocyte glycoprotein peptide. It was found that prophylactic administration of iguratimod from the time of immunization could significantly reduce the clinical severity of acute and chronic EAE. Pathologically, iguramod treatment significantly reduced the demyelination and infiltration of CD3 T, F4/80 and CD169 cells in the spinal cord compared with vector treatment, and inhibited the activation of macrophages/microglia in the acute and chronic phases. Iguratimod treatment after clinical symptoms can significantly improve the clinical severity of chronic EAE, reduce demyelination, helper T cell (Th) 1/Th17 cell infiltration, macrophage/microglia activation, and nuclear factor (NF) -κB p65 and cyclooxygenase-2 expression in the spinal cord. *In vitro*, Iguratimod treatment inhibited nuclear translocation of NF-κB p65 and down-regulated pro-inflammatory responses in macrophages and microglia. Their results suggest that Iguratimod improves acute and chronic EAE by inhibiting inflammatory cell infiltration and immune cell activation, partly by inhibiting NF-κB p65, supporting the therapeutic potential of the drug not only for acute but also for chronic MS (55). Studies have found that iguratimod not only exhibits selective MIF inhibition *in vitro* and *in vivo*, but also has an additive effect with glucocorticoids. In addition, we found that Iguratimod synergized with glucocorticoids to reduce experimental autoimmune encephalitis, which is a model of multiple sclerosis. The work identified iguratimod as a valuable new candidate for drug reuse for MIF-related diseases (including multiple sclerosis) (65).

### 3.8 Iguratimod in the treatment of connective tissue disease-related interstitial lung disease

Connective tissue-related interstitial lung disease, also known as CTD-ILD, refers to systemic lupus erythematosus, myositis, dermatomyositis, systemic sclerosis, scleroderma, rheumatoid arthritis, Sjogren ‘s syndrome and other connective tissue diseases caused by lung involvement, resulting in disease (168). As a common clinical autoimmune disease involving multiple organs and multiple systems, the lesions often involve the respiratory system (169). The causes of lung involvement may be related to immune response, triggering inflammation, pulmonary capillary endothelial injury, regulation of growth factors and cytokines (168). The time and degree of various connective tissue diseases involving the respiratory system are different. About one-fifth of patients, the typical lesions of connective tissue diseases are later than connective tissue disease-related interstitial lung diseases, and the lung manifestations are years or months, especially rheumatoid arthritis, dermatomyositis, polymyositis, and systemic lupus erythematosus (170). Studies have shown that ILD is associated with significant morbidity and mortality in almost all CTD patients (171). This highlights the urgent need for effective treatment strategies in this patient group.

Shao et al. studied the effects of Iguratimod on bleomycin-induced interstitial lung disease and related tumor necrosis factor -α (TNF-α) signaling pathways in mice and alveolar epithelial cells A549. They found that Iguratimod reduced lung inflammation and fibrosis and the expression of fibrosis-related genes, such as collagen I, α- smooth muscle actin (α-SMA), and bleomycin-induced MMP-2. Iguratimod inhibits epithelial - mesenchymal transition, as evidenced by decreased expression of E- cadherin but increased expression of vimentin. Iguratimod reduces TNF-α production in a mouse model of pulmonary fibrosis and in A549 cells cultured *in vitro*. Iguratimod decreased IL-6 production and STAT3 phosphorylation. In summary, Iguratimod -mediated anti-fibrotic effects are associated with inhibition of TNF-α and NF-Κb (172). Iguratimod attenuated bleomycin-induced PF in mice and improved pathological changes in mouse lungs. Furthermore, Iguratimod significantly attenuated TGF-β1-induced increases in mRNA expression in collagens I and III, thereby reducing lung function impairment, as well as α-SMA, Smad2 and Smad3 phosphorylation, fibronectin expression, and F-actin Actin filament formation, thereby attenuating FMT by inhibiting the Smad3 pathway (157). Iguratimod can reduce BLM-induced pulmonary fibrosis, and the anti-fibrotic effect of Iguratimod is partially mediated through the inhibition of MMP-9, suggesting that Iguratimod may be an effective therapeutic strategy for pulmonary fibrosis. Liu et al. found that Iguratimod produced favorable therapeutic effects by affecting inflammatory infiltration and collagen deposition. Furthermore, Iguratimod can inhibit the EMT process and NLRP3 inflammasome activation, reduce ROS production to improve pulmonary fibrosis, which may provide new insights for the further application of Iguratimod in interstitial pulmonary fibrosis (156). Han et al. found Iguratimod improved lung function in mice, including compliance (Crs), tissue damping (G), static compliance (Cst), inspiratory capacity (IC), elasticity (Ers), tissue elasticity (H) and respiratory system Resistance (Rrs). Iguratimod reduced BLM-induced changes in lung fibrosis and lung inflammation, as shown by histological examination. Iguratimod decreased the expression of immunoglobulin IgG and type I collagen in mice with BLM-induced lung fibrosis by suppressing B cell and immunoglobulin production, and also delayed the deterioration of imaging changes (158). Shao et al. found that Iguratimod reduced lung inflammation and fibrosis and the expression of fibrosis-related genes, such as collagen I, α-smooth muscle actin (α-SMA) and bleomycin-induced matrix metalloproteinase-2 (MMP-2). Iguratimod inhibits epithelial-mesenchymal transition, as evidenced by decreased expression of E-cadherin but increased expression of vimentin (172).

### 3.9 Iguratimod in other related inflammatory diseases

In terms of anti-tumor, the purpose of a study was to evaluate the anti-angiogenic and anticancer properties of iguramod (an anti-inflammatory drug similar to rheumatoid arthritis) against hepatocellular carcinoma. *In vitro* angiogenesis assay showed that the angiogenesis of the Iguratimod group was significantly lower than that of the control group (p = 0.013). The maximum tumor diameter (p = 0.036), tumor number (p = 0.011) and serum interleukin-8 concentration (p = 0.036) were significantly lower than those in the control group. Iguratimod can inhibit hepatocellular carcinogens by inhibiting the production of interleukin-8 in rat models (173). The findings suggest that iguramod can attenuate bone destruction by partially reducing IL-6 expression in an NF-κB-dependent manner, with little effect on tumor proliferation and invasion (174). OA is a degenerative disease that mainly damages articular cartilage. Peng Yangqianzi et al. found that iguratimod can protect rat degenerated chondrocytes by regulating the Wnt/β-catenin signaling pathway (175). The study of shu et al. showed that high-dose iguratimod had a direct inhibitory effect on the proliferation, differentiation and expression of OA-FLS, and regulated the immune function of OA synovitis through this mechanism (176). In the clinical study of 24 weeks after knee OA arthroscopy, Wu Jingxiong et al. found that the application of iguratimod was superior to diclofenac in terms of stress level, immune level and knee joint function (177). MM is an incurable malignant plasma cell proliferative disease. Its treatment often starts from inhibiting the production of immunoglobulins and cytokines and regulating bone metabolism. Gao Qiuying et al. confirmed *in vitro* that iguratimod can block the G0/G1 phase of MM cells by down-regulating CDK2 protein levels, thereby inhibiting the proliferation of myeloma cell line U226; it can also induce apoptosis of RPMI822 cells by up-regulating the expression of Bax protein that promotes apoptosis, and inhibit the activation of Notch pathway. The treatment of MM may therefore obtain new drug targets (178).

In addition, Wu et al. found that iguratimod could enhance the inhibitory effect of mitomycin C on the viability of esophageal cancer Eca109 cells and eventually lead to apoptosis. Bone metastasis of malignant tumors can increase osteoclast activity and lead to hypercalcemia (179). Sun Yue applied iguratimod to the study of breast cancer bone metastasis rats, and found that iguratimod could significantly inhibit the activation of osteoclasts (180). In addition, in inflammatory myopathy, Li et al. found that FVC % pred, FEV1% pred, DLCO/VA % pred, VC % pred, TLC % pred after treatment were higher than those before treatment. After treatment, PaCO2 was lower than that before treatment, and PaO2 was higher than that before treatment. There were significant differences in the incidence of grid shadow, ground glass shadow and interlobular septal thickening before and after treatment. The levels of serum TNF-α, IL-2, IL-6 and KL-6 after treatment were lower than those before treatment. The 6 min walking distance after treatment was longer than that before treatment. No serious adverse reactions occurred before and after treatment (181). In the treatment of Behcet ‘s disease, Wang et al. were divided into 25 cases in the Chinese and Western medicine group and 25 cases in the western medicine group according to the random number table method. The Chinese and Western medicine group was treated with modified Huatan Quyu decoction combined with iguratimod tablets, and the western medicine group was treated with iguratimod tablets. The clinical efficacy, C-reactive protein level, immunoglobulin A level and adverse drug reactions were compared between the two groups after 2 months of treatment. Results After treatment, the levels of serum C-reactive protein and immunoglobulin A in the two groups were lower than those before treatment (P < 0.01). The levels of C-reactive protein and immunoglobulin A in the Chinese and Western medicine group were significantly lower than those in the Western medicine group. The clinical efficacy of the Chinese and western medicine group was higher than that of the western medicine group, and the difference was statistically significant. The incidence of adverse reactions in the Chinese and western medicine group was lower than that in the western medicine group (182).

## 4 Prospective

Based on the current research reports, it can be found that: (1) Compared with drugs such as MTX and leflunomide, iguratimod, as a new type of DMARDs, has a new mechanism of action and clinical features. Its clinical trials have been extended to multiple inflammatory diseases and immune diseases (such as RA, knee osteoarthritis, IgG4-RD, systemic lupus erythematosus and lupus nephritis, PSS, CTD-ILD, erosive lichen planus, etc.). (2) Iguratimod has a clear immune-inflammation regulation effect, comprehensive bone protection effect, and anti-fibrosis effect. Its mechanism of action is comprehensive, taking into account efficacy and safety, and is suitable for combined treatment and long-term maintenance treatment of RA. (3) Iguratimod has various mechanisms of action and has a comprehensive immune regulation effect. Studies have shown that iguratimod can regulate the immune balance mediated by T cells and related inflammatory factors by regulating the number of helper T cells Th1 and Th17, Tfh cells and Treg cells. It can also inhibit the differentiation process of B cells into plasma cells, thereby inhibiting the production of autoantibodies. In addition, iguratimod can also inhibit the secretion of TNF-α, IL-1β, IL-6, IL-17 and other cytokines, suggesting that it has a strong anti-inflammatory effect. (4) Iguratimod has both anti-inflammatory and anti-fibrotic effects. It can not only effectively control the symptoms of rheumatic immune diseases (such as RA, PSS, etc.), but also delay the progression of ILD. The mechanism is to reduce ROS production by inhibiting EMT process and NLRP3 inflammasome activation, thereby improving pulmonary fibrosis. This provides a new idea for the further application of iguratimod in interstitial pulmonary fibrosis, and provides a new choice for clinical rheumatic immune diseases and related ILD patients or CTD-ILD patients. (5) Clinical evidence-based evidence comprehensively shows that the long-term response of iguratimod monotherapy is better than that of MTX, and the safety is good. Secondly, many patients with ERA and UA are in the early stage of the disease and the disease activity is low, and a good therapeutic response can be achieved with Iguratimod monotherapy. In addition, the performance of Iguratimod in delaying joint damage is also impressive. Previous basic research has found that iguratimod has a bone metabolism regulating effect, which is consistent with the clinically demonstrated bone protection effect. These all suggest the possibility of iguratimod as the preferred first-line treatment for RA, and provide a new option for treatment-naïve RA patients who are intolerant to MTX. In the future, we look forward to more research to further improve the clinical application of iguratimod, and ultimately benefit patients with rheumatic immune diseases. Due to the unique pharmacological mechanism of iguratimod, it has also shown initial efficacy in other rheumatic immune diseases (such as systemic lupus erythematosus, PSS, etc.), and it is believed that more patients with rheumatic diseases will benefit from it in the future.

In summary, there have been many clinical studies on iguratimod in China, but there are shortcomings such as small sample size, inconsistent disease indicators, and short research cycle. Autoimmune diseases are mostly chronic progressive diseases. At present, there is no radical method, and the treatment is mainly to control the disease. Therefore, it is recommended to conduct long-term safety assessment. Multi-center clinical trials over 3 years or even longer can provide more reliable safety support for the long-term application of the drug. In 2015, the drug was included as a recommended drug for clinical guidelines by the Asia-Pacific Rheumatism Alliance (APLAR), but most of the subsequent studies were concentrated in East Asia. More extensive international cooperation in clinical research can make iguratimod an effective choice for patients with rheumatic bone diseases and autoimmune diseases worldwide. In the future, more research is still needed for its wider indications and better combination therapy.

## Author contributions

ZL, LZ, QH, KY and LS are responsible for the study concept and design. ZL, LZ, QH, KY, WX, XR,YD, HC are responsible for the literature collection; LZ drafted the paper; LS supervised the study. All authors contributed to the article and approved the submitted version.
