# Supplementary material for: Research progress on the clinical application and mechanism of iguratimod in the treatment of autoimmune diseases and rheumatic diseases
Source: Front Immunol. 2023 Sep 21;14:1150661. doi: 10.3389/fimmu.2023.1150661 (PMC10552782; doi:10.3389/fimmu.2023.1150661)
Supplement: Supplementary file 1 [file Table_1.docx]

Table S1 RCT of iguratimod in the treatment of RA

| **RCTs** | **Intervention** | | **Relevant outcomes** | **Duration** |
| --- | --- | --- | --- | --- |
|  | **Trial group** | **Control group** |  |  |
| Bi et al. 2019 [1] | IGU 25mg Bid+MTX 10 mg once a week | MTX 10 mg once a week +Leflunomide 20mg Qd | DAS28, adverse events | 12 weeks |
| Chen et al. 2018 [2] | IGU 25mg Bid+MTX 10 mg once a week | MTX 10 mg once a week | CRP, adverse events | 24 weeks |
| Deng et al. 2017 [3] | a: IGU 25mg Bid+MTX 10 mg once a week; b: IGU 25mg Bid | MTX 10 mg once a week +Leflunomide 20mg Qd | DAS28, ESR, CRP, RF, adverse events | 48 weeks |
| Duan et al. 2015 [4] | IGU 25mg Bid+MTX 10 mg once a week at the beginning, gradually increase to 12.5mg within 4 weeks | MTX 10 mg once a week at the beginning, gradually increase to 12.5mg within 4 weeks | ESR, CRP, DAS28, adverse events | 24 weeks |
| Fan et al. 2020 [5] | IGU 25mg Bid+MTX 10 mg once a week at the beginning; 12.5 mg once a week after 2 weeks; 15 mg once a week after 4 weeks | MTX 10 mg once a week at the beginning; 12.5 mg once a week after 2 weeks; 15 mg once a week after 4 weeks | DAS28 | 24 weeks |
| Hara et al. 2007 [6] | IGU 25 mg for the first 4 weeks and 50 mg for the subsequent 24 weeks | placebo | CRP, ESR, adverse events | 28 weeks |
| Hara et al. 2014 [7] | IGU 25mg Qd for the first 4 weeks of the extension period 25 mg Bid for the subsequent 20 week+MTX 6-8 mg once a week | MTX 6-8 mg once a week+placebo | ACR20, ACR50, ACR70, CRP, RF, DAS28, adverse events | 24 weeks |
| Hu et al. 2014 [8] | IGU 25mg Bid | MTX 10 mg once a week | DAS28, ACR20, adverse events | 24 weeks |
| Ju et al. 2020 [9] | IGU 25mg Bid+MTX 10 mg once a week | MTX 10 mg once a week | DAS28, ESR, CRP, RF | 24 weeks |
| Li et al. 2016 [10] | IGU 25mg Qd+MTX 7.5-10 mg once a week | MTX 7.5-10 mg once a week+Tripterygium glycosides 20mg Bid | DAS28, ESR, CRP, adverse events | 12 weeks |
| Li et al. 2019 [11] | IGU 25mg Bid+MTX 15 mg once a week | MTX 15 mg once a week | Adverse events | 15 weeks |
| Li et al. 2020 [12] | IGU 25mg Bid+MTX 10 mg once a week | MTX 10 mg once a week+Adalimumab 40mg once every 2 weeks | DAS28 | 24 weeks |
| Lü et al. 2008 [13] | a: IGU 25 mg Qd; b: 25mg Bid | Placebo | ACR20, ACR50, ACR70, ESR, CRP, RF, adverse events | 24 weeks |
| Lu et al. 2009 [14] | a: IGU 25 mg for the first 4 weeks and 50 mg for the subsequent 20 weeks; b: IGU 25mg Bid | MTX 10 mg/week for the first 4 weeks and 15 mg/week for the subsequent 20 weeks | ACR20, ACR50, ACR70, ESR, CRP, RF, adverse events | 24 weeks |
| Meng et al. 2015 [15] | IGU 25mg Bid+MTX 10 mg once a week | MTX 10 mg once a week+Leflunomide 10mg Qd | DAS28, ACR20, ACR50, ACR70, adverse events | 16 weeks |
| Meng et al. 2016 [16] | IGU 25mg Bid+MTX 15 mg once a week | MTX 15 mg once a week | DAS28, adverse events | 16 weeks |
| Meng et al. 2017 [17] | IGU 25mg Bid+MTX 10 mg once a week | MTX 10 mg once a week | RF, CRP, adverse events | 12 weeks |
| Mo et al. 2015 [18] | IGU 25mg Bid+MTX 15 mg once a week | MTX 15 mg once a week | ACR20, ACR50, ACR70, ESR, CRP, RF, adverse events | 12 weeks |
| Mo et al. 2018 [19] | IGU 25mg Bid+MTX 10 mg once a week | MTX 10 mg once a week+Tripterygium glycosides 20mg Bid | DAS28, ESR, CRP, CCP, RF, adverse events | 12 weeks |
| Qi et al. 2019 [20] | IGU 25mg Bid+MTX 7.5 mg once a week at the beginning, gradually increase to 10mg within 4 weeks | MTX 7.5 mg once a week at the beginning,Gradually increase to 10mg within 4 weeks | ACR20, ACR50, ACR70, ESR, CRP, adverse events | 24 weeks |
| Shi et al. 2015 [21] | IGU 25mg Bid+MTX 10 mg once a week at the beginning;12.5 mg twice a week after 4 weeks | MTX 10 mg once a week at the beginning;12.5 mg twice a week after 4 weeks | DAS28, ESR, CRP, ACR20, ACR50, ACR70, adverse events | 24 weeks |
| Tian et al. 2017 [22] | IGU 25mg Bid+MTX 10 mg once or twice a week | MTX 10 mg once or twice a week | DAS28, ESR, CRP, adverse events | 24 weeks |
| Tian et al. 2020 [23] | IGU 25mg Bid+MTX 10 mg once a week | MTX 10 mg once a week +Leflunomide 20mg Qd | DAS28, ESR, CRP, RF, adverse events | 52 weeks |
| Wang et al. 2019 [24] | IGU 25mg Bid+MTX 15 mg once a week | MTX 15 mg once a week | CRP, RF, ESR, DAS28 | 24 weeks |
| Xia et al. 2016 [25] | a: IGU 25mg Bid+MTX 10 mg once a week; b: IGU 25mg Bid | MTX 10 mg once a week | ESR, CRP | 24 weeks |
| Xia et al. 2020 [26] | IGU 25mg Bid+MTX 7.5 mg once a week at the beginning, increase by 2.5mg per week, with a final dose of 15mg | MTX 7.5 mg once a week at the beginning, increase by 2.5mg per week, with a final dose of 15mg+Tripterygium glycosides 1-1.5mg/kg | ESR, CRP | 12 weeks |
| Xie et al. 2018 [27] | IGU 25mg Bid+MTX 10 mg once a week at the beginning; 12.5 mg twice a week after 2 weeks; 15 mg once a week after 4 weeks | MTX 10 mg once a week at the beginning; 12.5 mg twice a week after 2 weeks; 15 mg once a week after 4 weeks | DAS28, adverse events | 16 weeks |
| Xiong et al. 2015 [28] | IGU 25mg Bid+MTX 10 mg once a week | MTX 10 mg once a week | RF, CRP, ESR, DAS28, adverse events | 24 weeks |
| Xiong et al. 2020 [29] | IGU 25mg Bid+MTX 10 mg once a week at the beginning; 12.5 mg twice a week after 2 weeks; 15 mg once a week after 4 weeks | MTX 10 mg once a week at the beginning; 12.5 mg twice a week after 2 weeks; 15 mg once a week after 4 weeks | Adverse events | 24 weeks |
| Xu et al. 2015 [30] | a: IGU 25mg Bid+MTX 7.5-20mg once a week; b: IGU 25mg Bid | MTX 7.5-20mg once a week | ESR, CRP, RF, adverse events | 48 weeks |
| Xu et al. 2017 [31] | IGU 25mg Bid+MTX 7.5-20mg once a week | MTX 7.5-20mg once a week | DAS28, ESR, CRP | 48 weeks |
| Yan et al. 2018 [32] | IGU 25mg Bid+MTX 10 mg once a week | MTX 10 mg once a week | Adverse events | 24 weeks |
| Zhao et al. 2016 [33] | a: IGU 25mg Bid+MTX 10 mg once a week; b: IGU 25mg Bid | MTX 15 mg once a week | ACR20, ACR50, ACR70, adverse events | 24 weeks |
| Zhao et al. 2017 [34] | a: IGU 25mg Bid; b: IGU 25mg Bid+MTX 10 mg once a week | MTX 10 mg once a week | ACR20, ACR50, ACR70, DAS28, ESR, CRP, RF, adverse events | 24 weeks |
| Zhao et al. 2018 [35] | IGU 25mg Bid+MTX 7.5 mg once a week | MTX 7.5 mg once a week | DAS28, CRP, adverse events | 12 weeks |

**Reference**

1. Bi WH. The effect of Iguratimod combined with methotrexate on serum VEGF levels in patients with rheumatoid arthritis and evaluation of the efficacy[D]. Inner Mongolia Medical University, 2019. (in chinese)
2. Chen J, Ding ZH, Liu J. Effects of Iguratimod combined with methotrexate on serum inflammatory factors and bone metabolism in patients with rheumatoid arthritis.Zhejiang Journal of Integrated Traditional Chinese and Western Medicine,28(2018):552-555. (in chinese)
3. Deng JX The effect of Iguratimod on the proliferation and migration of fibroblast-like synovial cells in rheumatoid arthritis and the clinical observation[D]. Southern Medical University,2017.(in chinese)
4. Duan XW, Zhang XL, Mao SY, Shang JJ, Shi XD. Efficacy and safety evaluation of a combination of iguratimod and methotrexate therapy for active rheumatoid arthritis patients: a randomized controlled trial. Clin Rheumatol. 34(2015):1513-9. doi: 10.1007/s10067-015-2999-6.
5. Fan ZX, Wu PC, Song MH, et al. The clinical efficacy of iguratimod combined with methotrexate in the treatment of rheumatoid arthritis. Journal of Clinical Rational Use.13(2020):81-83, (in chinese)
6. Hara M, Abe T, Sugawara S, Mizushima Y, Hoshi K, Irimajiri S, Hashimoto H, Yoshino S, Matsui N, Nobunaga M, Nakano S. Efficacy and safety of iguratimod compared with placebo and salazosulfapyridine in active rheumatoid arthritis: a controlled, multicenter, double-blind, parallel-group study. Mod Rheumatol. 2007;17(1):1-9. doi: 10.1007/s10165-006-0542-y. Epub 2007 Feb 20. PMID: 17278015.
7. Hara M, Ishiguro N, Katayama K, Kondo M, Sumida T, Mimori T, Soen S, Nagai K, Yamaguchi T, Yamamoto K; Iguratimod-Clinical Study Group. Safety and efficacy of combination therapy of iguratimod with methotrexate for patients with active rheumatoid arthritis with an inadequate response to methotrexate: an open-label extension of a randomized, double-blind, placebo-controlled trial. Mod Rheumatol. 24(2014):410-8. doi: 10.3109/14397595.2013.843756.
8. Hu Huiling. Efficacy and safety observation of iguratimod in the treatment of rheumatoid arthritis[D]. Suzhou University, 2014.
9. Ju YJ, Guo DB, Chen H, et al. Evaluation of the clinical efficacy of methotrexate and ilamod in the treatment of refractory rheumatoid arthritis. China Modern Doctor, 058(2020):106 -109. (in chinese)
10. Li J, Chen QP, Liu JY, et al. The treatment of 84 cases of senile rheumatoid arthritis with the combination of iguratimod and methotrexate. Shaanxi Medical Journal, 45(2016):120-121. (in chinese)
11. Li Lianju, Wang Jingxu, Li Xiaoli. A clinical study on the treatment of early rheumatoid arthritis by iguratimod combined with methotrexate. China Pharmaceuticals,28(2019):63-65. (in chinese)
12. Li CH, H WH. The effect of irammod combined with methotrexate in the treatment of rheumatoid arthritis with peripheral blood nuclear factor kappa B receptor activator ligand and bone protective factor.Chinese Medicines and Clinics,20(2020):2981-2983.(in chinese)
13. Lü LJ, Teng JL, Bao CD, Han XH, Sun LY, Xu JH, Li XF, Wu HX. Safety and efficacy of T-614 in the treatment of patients with active rheumatoid arthritis: a double blind, randomized, placebo-controlled and multicenter trial. Chin Med J (Engl). 2008 Apr 5;121(7):615-9. PMID: 18466681.
14. Lu LJ, Bao CD, Dai M, Teng JL, Fan W, Du F, Yang NP, Zhao YH, Chen ZW, Xu JH, He PG, Wu HX, Tao Y, Zhang MJ, Han XH, Li XF, Gu JR, Li JH, Yu H. Multicenter, randomized, double-blind, controlled trial of treatment of active rheumatoid arthritis with T-614 compared with methotrexate. Arthritis Rheum. 2009 Jul 15;61(7):979-87. doi: 10.1002/art.24643. PMID: 19565542.
15. Meng DY, Wang GR, Pan WY, et al. Short-term clinical efficacy of methotrexate combined with Iguratimod on refractory rheumatoid arthritis. Chinese Journal of Clinical Research,28(2015 ): 40-42. (in chinese)
16. Meng DY, Pan WY, Li J, Li H, Li F, Liu SS, Li YS, Cheng YL. The effect of methotrexate combined with Ilamud in the treatment of refractory rheumatoid arthritis.China Medical Herald, 13(2016):137-141. (in chinese)
17. Meng Y,Li MY,Rode M,Zhang XY,Luo L.Clinical study on the treatment of senile rheumatoid arthritis with Iguratimod tablets combined with methotrexate tablets.Chinese Journal of Clinical Pharmacology,33(2017):1098-1101.
18. Mo H, Ma SB.Clinical study on the treatment of active rheumatoid arthritis with Iguratimod combined with methotrexate.Internal Medicine,10(2015):156-159. (in chinese)
19. Mo ML, Tang DX, Zhang J, et al. A randomized controlled trial of methotrexate combined with Iguratimod in the treatment of active rheumatoid arthritis. Journal of Fujian Medical University, 52(2018) :40-43. (in chinese)
20. Qi DX, Liu Y, Huang DH. Study on the efficacy and safety of methotrexate and isilamod in the treatment of rheumatoid arthritis. Chinese Journal of Drug Evaluation, 036(2019):217-220. (in chinese)
21. Shi XD, Zhang XL, Duan XW. Efficacy and safety of methotrexate combined with iguratimod in the treatment of active rheumatoid arthritis.Journal of Nanchang University (Medical Edition),55(2015):33- 36+47.（in chinese）
22. Tian JW, Tao PF. The effect of Iguratimod combined with methotrexate on serum M-CSF, IL-6, IL-8 and bone metabolism in patients with rheumatoid arthritis.Hainan Medicine,28(2017):391-394. (in chinese)
23. Tian XP, Liu SY, Li Q, Bi LQ, Kong XD, Zhao DB, Hu SX, Zhang ZL, Lin J, Zhang FC. Efficacy and safety of ellamod or leflunomide combined with methotrexate in the treatment of active rheumatoid arthritis Sexual comparison: a multicenter randomized double-blind double-simulation controlled clinical study.Chinese Journal of Rheumatology,24(2020):148-158. (in chinese)
24. Wang Longhui, Liu Shengxing, Chen Rong, et al. Clinical study on the treatment of rheumatoid arthritis with methotrexate tablets combined with Iguratimod tablets. Chinese Journal of Clinical Pharmacology,35(2019):231-234 . (in chinese)
25. Xia Z, Lyu J, Hou N, Song L, Li X, Liu H. Iguratimod in combination with methotrexate in active rheumatoid arthritis : Therapeutic effects. Z Rheumatol. 75(2016):828-833. English. doi: 10.1007/s00393-015-1641-y.
26. Xia NN, Chen ZF, Zhang WF. Observation on the effects of Iguratimod and Tripterygium Glycosides in the treatment of rheumatoid arthritis. Practical Integrated Traditional Chinese and Western Medicine.20(2020):76-77. (in chinese)
27. Xie Li, Zou QH, Shi Y, Cheng X, Fang YF. The effect of Iguratimod combined with MTX on IL-1, serum TNF-α and VEGF levels in patients with refractory rheumatoid arthritis.Guizhou Medicine, 42(2018):831-832. (in chinese)
28. Xu YM, Fan SN, Zou L. Study on the effect of iguratimod combined with methotrexate treatment on anti-cyclic guanidine peptide antibodies and other indicators in patients with rheumatoid arthritis. Journal of Clinical Internal Medicine,32 (2015):833-835. (in chinese)
29. Xiong Meili, Geng Guanghui. The clinical efficacy of methotrexate combined with iguratimod on active rheumatoid arthritis.Henan Medical Research,2020,29(2020):93-95.(in chinese)
30. Xu Baijie, Mo Shouqi, Xue Xiaoqian. Clinical study of methotrexate combined with Ilamud in the treatment of rheumatoid arthritis. Journal of Clinical Medicine in Practice,19(2015):120-122. .(in chinese)
31. Xu LM, Yuan M, Liu YF, Sun HX, Liu LN, Shi YJ, Li CX.Clinical observation on the treatment of rheumatoid arthritis with Iguratimod combined with methotrexate.Guide to Chinese Medicine, 15(2017): 47-48. (in chinese)
32. Yan XZ, Wang FL. Effect of Iguratimod combined with methotrexate on serum-related cytokines and bone metabolism in patients with rheumatoid arthritis.Journal of Changchun University of Traditional Chinese Medicine, 34(2018):369-372 . (in chinese)
33. Zhao WM,Yao DY,Huo HS,Qin CM,Wei QJ,Sun K.Clinical study of Iguratimod in the treatment of active rheumatoid arthritis.Chinese Journal of Postgraduates of Medicine,39(2016):450 -452.(in chinese)
34. Zhao L , Jiang Z , Zhang O , et al. Analysis of efficacy and safety of treatment of active rheumatoid arthritis with iguratimod and methotrexate[J]. Biomedical Research. 2017. 28(5), 2353-2359
35. Zhao HN, Hao XJ. The clinical effect of Iguratimod combined with methotrexate in the treatment of rheumatoid arthritis. Clinical Medical Research and Practice, 003(2018):44-45. (in chinese)
