# Supplementary material for: Research progress on the clinical application and mechanism of iguratimod in the treatment of autoimmune diseases and rheumatic diseases
Source: Front Immunol. 2023 Sep 21;14:1150661. doi: 10.3389/fimmu.2023.1150661 (PMC10552782; doi:10.3389/fimmu.2023.1150661)
Supplement: Supplementary file 2 [file Table_2.docx]

Table S2 RCT of iguratimod in the treatment of pSS

| **RCTs** | **Intervention** | | **Relevant outcomes** | **Duration** |
| --- | --- | --- | --- | --- |
|  | **Trial group** | **Control group** |  |  |
| Bai et al. 2019 [1] | Iguratimod 25 mg Bid | Methylprednisolone 8 mg Qd + HCQ 200 mg Bid + Leflunomide 50mg Qd | ESSPRI, ESSDAI, RF, ESR, Adverse events | 12 weeks |
| Chen et al. 2022 [2] | Iguratimod 25 mg Bid+Total Glucosides of Paeony 0.6g Tid + HCQ 0.2g Bid | Total Glucosides of Paeony 0.6g Tid + HCQ 0.2g Bid | ESSPRI, ESSDAI, ESR, RF | 12 weeks |
| Gu 2020 [3] | Iguratimod 25 mg Bid | Prednisone 8 mg Qd + HCQ 200 mg Bid | RF, Adverse events | 12 weeks |
| Gu 2022 [4] | Iguratimod 25 mg Bid | Methylprednisolone 8 mg Qd + HCQ 200 mg Bid | ESSPRI, adverse events | 2 weeks |
| Jia 2020 [5] | Iguratimod 25 mg Bid | Methylprednisolone 8 mg Qd + HCQ 200 mg Bid | ESSPRI, ESSDAI, ESR, RF, adverse events | 16 weeks |
| Jiang et al. 2014 [6] | Iguratimod 25 mg Bid | Prednisone 5-10 mg Qd + HCQ 200 mg Bid+Bromoethylsine 16mg Bid | ESSPRI, ESSDAI, Schirmer’s test, Adverse events | 12 weeks |
| Jiang et al. 2016 [7] | Iguratimod 50 mg Qd | Prednisone 8 mg Qd + HCQ 200 mg Bid | RF, ESR, Adverse events | 12 weeks |
| Jiang et al. 2020 [8] | Iguratimod 50 mg Qd | Prednisone 10 mg, hydroxychloroquine (HCQ) 400 mg, new hydrochloride bromine ethyl Qd | EULAR Sjögren’s syndrome patient-reported index (ESSPRI), ESSDAI, Schirmer’s test, Adverse events | 12 weeks |
| Jiang et al. 2021 [9] | Iguratimod 25 mg Bid+ Chere Cunjing Granules | Chere Cunjing Granules (Traditional Chinese Medicine) | ESSPRI, ESSDAI, ESR, CRP, adverse events | 12 weeks |
| Li et al. 2018 [10] | Iguratimod 25 mg Bid | Prednisone 8 mg Qd + HCQ 200 mg Bid | ESSPRI, RF, ESR, Adverse events | 12 weeks |
| Li et al. 2020 [11] | Iguratimod 25 mg Bid | Prednisone 8 mg Qd + HCQ 200 mg Bid | ESSPRI, ESR, Adverse events | 12 weeks |
| Liang et al. 2021 [12] | Iguratimod 25 mg Bid + Methylprednisolone 8 mg | Methylprednisolone 8 mg Qd + HCQ 200 mg Bid | ESSDAI, ESSPRI, ESR, CRP, adverse events | 16 weeks |
| Lu and Zhang 2021 [13] | Iguratimod 25 mg Bid+ HCQ 0.2g Bid | HCQ 0.2g Bid | ESR, RF, adverse events | 12 weeks |
| Luo et al. 2018 [14] | Iguratimod 25 mg Bid | Prednisone 8 mg Qd + HCQ 200 mg Bid | ESR, RF, adverse events | 12 weeks |
| Miu 2018 [15] | Iguratimod 25 mg Bid+Total Glucosides of Paeony 0.6g Tid + HCQ 0.2g Bid | Total Glucosides of Paeony 0.6g Tid + HCQ 0.2g Bid | ESR, CRP, Adverse events | 12 weeks |
| Rao et al. 2022 [16] | Iguratimod 25 mg Bid | Methylprednisolone 4 mg Qd + HCQ 200 mg Bid | Schirmer’s test, ESR, RF | 12 weeks |
| Shao et al. 2020 [17] | Iguratimod 25mg Bid | Placebo | ESSPRI, ESR, ESSDAI, Adverse events | 24 weeks |
| Wang et al. 2019 [18] | Iguratimod 25 mg Bid + Total Glucosides of Paeony 0.6g Bid + HCQ 0.1g Bid | Total Glucosides of Paeony 0.6g Bid + HCQ 0.1g Bid | ESSPRI, ESSDAI, Schirmer’s test, ESR, RF, Adverse events | 12 weeks |
| Wei 2019 [19] | Iguratimod 25 mg Bid + Methylprednisolone 8 mg | Methylprednisolone 8 mg Qd + HCQ 200 mg Bid | ESR, adverse events | 12 weeks |
| Xia et al. 2017 [20] | Iguratimod 25 mg Bid + Methylprednisolone | HCQ 200mg Bid + Methylprednisolone | ESR, RF | 12 weeks |
| Xie et al. 2020 [21] | Iguratimod 25 mg Bid+Total Glucosides of Paeony 0.6g Tid + HCQ 0.2g Bid | Total Glucosides of Paeony 0.6g Tid + HCQ 0.2g Bid | ESR, CRP, Schirmer’s test, Adverse events | 24 weeks |
| Xu et al. 2017 [22] | Iguratimod 25 mg Bid | Prednisone 8 mg Qd + HCQ 200 mg Bid | ESSPRI, ESSDAI, ESR, RF, Schirmer’s test | 12 weeks |
| Yu 2020 [23] | Iguratimod 25 mg Bid | Methylprednisolone 8 mg Qd + HCQ 200 mg Bid | ESR, RF | 12 weeks |
| Zhang 2019 [24] | Iguratimod 25 mg Bid + Methylprednisolone 8 mg | Methylprednisolone 8 mg Qd + HCQ 200 mg Bid | ESSPRI, ESSDAI, Schirmer’s test | 12 weeks |
| Zhang and Shen 2019 [25] | Iguratimod 25 mg Bid + Methylprednisolone 8 mg | Methylprednisolone 8 mg Qd + HCQ 200 mg Bid | ESSPRI, ESSDAI, ESR, RF, Schirmer’s test, adverse events | 12 weeks |
| Zhang et al. 2019 [26] | Iguratimod 25 mg Bid | Prednisone + HCQ + olfaction | FVC, maximum mid-expiratory flow (MMF), ESR, adverse events | 20 weeks |
| Zhao 2019 [27] | Iguratimod 25 mg Bid | Prednisone 8 mg Qd + HCQ 200 mg Bid | RF, ESR, Adverse events | 12 weeks |
| Zhao 2020 [28] | Iguratimod 25 mg Bid + Basic therapy | HCQ 200 mg Bid + Basic therapy | ESR, RF, adverse events | Unkown |
| Zhuang 2020 [29] | Iguratimod 25 mg Bid + Methylprednisolone 8 mg | Methylprednisolone 8 mg Qd + HCQ 200 mg Bid | ESR, RF | 12 weeks |

**Reference**

1. Bai Jie, Jiao Yingwei. Observation on the clinical effect of Iramod in the treatment of primary Sjogren’s syndrome. Shanxi Medical Journal, 2019, 48(14): 1724-1726.
2. Chen Yuling, Shen Peng, Sun Dandan. Analysis of curative effect of iguratimod in the treatment of elderly patients with pSS and its effect on B cell activity and secretion level of immunoglobulin G.Medical Theory and Practice,2022,35(02) :244-246.DOI:10.19381/j.issn.1001-7585.2022.02.025. (in chinese)
3. Gu Jing.Clinical effect of methylprednisolone combined with Ilamod in the treatment of elderly patients with primary Sjogren’s syndrome.Chinese National Health Medicine,2020,32(18):1-2+5.
4. Gu Jianmei. Clinical effect of methylprednisolone combined with iguratimod in the treatment of primary Sjögren's syndrome and its influence on the level of immunoglobulin . Contemporary Medicine, 2022, 28(08): 158-160. (in chinese)
5. Jia Xuqiang. Analysis of the effect of iguratimod combined with hydroxychloroquine in the treatment of Sjögren's syndrome . Gansu Science and Technology, 2020,36(18):106-108. (in chinese)
6. Jiang Wei, Zhao Yi, Lin Hui, Liu Yi, Chen Jin. Observation on the curative effect of Iramod in the treatment of primary Sjogren’s syndrome.Western Medicine, 2014,26(06):719-721 +724.
7. Jiang Dexun,BaiYunjing,ZhaoLiping,ZhangYifan,ChenZhu.Observation on the clinical effect of Iramod combined treatment of primary Sjogren’s syndrome.Clinical Misdiagnosis and Mistreatment,2016,29(08):90-93.
8. Jiang W, Zhang L, Zhao Y, He X, Hu C, Liu Y. The efficacy and mechanism for action of iguratimod in primary Sjögren's syndrome patients. Int Ophthalmol. 2020 ,40(11):3059-3065.
9. Jiang Huihui. Clinical observation of Shure Cunjin granules combined with iguratimod in the treatment of primary Sjögren's syndrome with high IgG[D]. Hunan University of Traditional Chinese Medicine, 2021. DOI: 10.27138/d.cnki.ghuzc.2021.000018.(in chinese)
10. Li Chuanjing,LiRui,LiuHanzhong,ChengChuanfang,ZhaoTao.Efficacy of methylprednisolone combined with ilamod in the treatment of primary Sjogren’s syndrome and its effect on immunoglobulin levels.China Pharmaceuticals,2018,27( 14): 35-37.
11. Li Rongrong,LongHong.Efficacy of methylprednisolone combined with Ilamod in the treatment of primary Sjogren’s syndrome and its effect on immunoglobulin levels.World Complex Medicine,2020,6(05):189-191.
12. Liang Zhuoyuan, Wei Feng, Ouyang Chujun, Feng Mingliang. Effects of iguratimod on the levels of ESR, CRP and immunoglobulin in patients with primary Sjögren's syndrome . Shanghai Medicine, 2021, 42(01): 32-35. (in chinese)
13. Lu Ting, Zhang Wei. Clinical efficacy of iguratimod combined with hydroxychloroquine sulfate in the treatment of primary Sjögren's syndrome . Journal of Clinical Rational Medicine, 2021, 14(31): 55-57. DOI: 10.15887/ j.cnki.13-1389/r.2021.31.018. (in chinese)
14. Luo Qiwen, Guo Dongmei, Yu Yangtao, Lin Jiahong. Efficacy and safety of iguratimod and hydroxychloroquine in the treatment of patients with Sjögren's syndrome . Clinical Research in Traditional Chinese Medicine, 2018,10(24):94-95. (in chinese)
15. Miao Yi. Observation of the changes of ESR, CRP, IgG, IgA, IgM levels in patients with primary Sjogren’s syndrome by Iramod. Electronic Journal of Clinical Medicine Literature, 2018, 5(95): 147+149.
16. Rao Yanting, Zhang Wei, Lu Ting, Xu Jingjing. Efficacy of iguratimod in the treatment of Sjögren's syndrome patients and its influence on immune function. Journal of Ningxia Medical University, 2022,44(02):152-156. DOI: 10.16050/j.cnki.issn1674-6309.2022.02.008. (in chinese)
17. Shao Q, Wang S, Jiang H, Liu L. Efficacy and safety of iguratimod on patients with primary Sjögren's syndrome: a randomized, placebo-controlled clinical trial. Scand J Rheumatol. 2020,29:1-10.
18. Wang Yanling, Zhao Futao, Ai Xiangyan, Liu Yang, Zhu Zhenhang. Observation on the efficacy and safety of Iramod in the treatment of primary Sjogren’s syndrome in the elderly. Geriatrics and Health Care, 2019, 25(02): 209-213.
19. Wei Donghui. Efficacy and safety of iguratimod and hydroxychloroquine in the treatment of Sjögren's syndrome . DOCTOR,2019,11:146-147. (in chinese)
20. Xia Zhongbin, Liu Ying, Meng Fanjin, et al. Analysis of clinical effect of iguratimod combined treatment on primary Sjögren's syndrome.China Health Nutrition, 2017, 27(034):263. (in chinese)
21. XieHuan,LiuYi,WangJian,ZengChunxue,ZhouYalu.Hydroxychloroquine sulfate combined with total glucosides of paeony and ilamod in the treatment of primary Sjogren’s syndrome.Western Medicine,2020,32(09):1358- 1362.
22. Xu Dong, Lv Xiaowei, Cui Peng, Ma Shangmin. Comparison of the efficacy and safety of iguratimod and hydroxychloroquine in the treatment of patients with Sjögren's syndrome . Journal of Difficult and Difficult Diseases, 2017,16(09):915-918. (in chinese)
23. Yu Weijing.Clinical analysis of Iramod in the treatment of primary Sjogren’s syndrome.Guide to Chinese Medicine,2020,18(06):150
24. Zhang Xiaoyan.Comparative observation of iguratimod and hydroxychloroquine in the treatment of patients with Sjögren's syndrome.Chinese Medicine Guide,2019,17(32):103.DOI:10.15912/j.cnki.gocm.2019.32.081. (in chinese)
25. Zhang Jing, Shen Siyao. Efficacy and mechanism of iguratimod in the treatment of Sjögren's syndrome . Shaanxi Medical Journal, 2019, 048(004):452-455. (in chinese)
26. Zhang Lihua, Zhong Miao, Li Qiao, Guo Xia. Efficacy evaluation of iguratimod in the treatment of Sjögren's syndrome complicated with interstitial pulmonary disease. China Modern Medicine Application, 2019,13(20):1-3.DOI :10.14164/j.cnki.cn11-5581/r.2019.20.001. (in chinese)
27. Zhao Ling. Observation on the effect of methylprednisolone combined with Iramud in the treatment of primary Sjogren’s syndrome. Chinese National Health Medicine, 2019,31(17):35-36.
28. Zhao Jianqing. Comparison of the efficacy and safety of iguratimod and hydroxychloroquine in the treatment of Sjögren's syndrome . Chinese Medicine and Clinical Medicine, 2020,20(21):3627-3629. (in chinese)
29. Zhuang Zhiyi. To explore the effect of iguratimod in the treatment of primary Sjögren's syndrome . Special Health 2020, 31, 100-101, 2020. (in chinese)
