# Supplementary material for: Research progress on the clinical application and mechanism of iguratimod in the treatment of autoimmune diseases and rheumatic diseases
Source: Front Immunol. 2023 Sep 21;14:1150661. doi: 10.3389/fimmu.2023.1150661 (PMC10552782; doi:10.3389/fimmu.2023.1150661)
Supplement: Supplementary file 3 [file Table_3.docx]

Table S3 RCT of iguratimod in the treatment of AS

| **RCTs** | **Intervention** | | **Relevant outcomes** | **Duration** |
| --- | --- | --- | --- | --- |
|  | **Trial group** | **Control group** |  |  |
| Bai et al. 2021 [1] | Iguratimod 25mg Bid + Sulfasalazine 1g Bid + Celecoxib 200mg Bid | Sulfasalazine 1g Bid + Celecoxib 200mg Bid | BASDAI, VAS, CRP, ESR, adverse events | 12 weeks |
| Li et al. 2021 [2] | Iguratimod 25mg Bid + Sulfasalazine 0.5 to 1g Bid + Thalidomide 50 to 200mg Qn | Sulfasalazine 0.5 to 1g Bid + Thalidomide 50 to 200mg Qn | BASDAI | 24 weeks |
| Lin et al. 2019 [3] | Iguratimod 25mg Bid + Sulfasalazine 1 g Bid. + methotrexate 10 mg once a week + NSAIDs | Sulfasalazine 1 g Bid. + methotrexate 10 mg once a week + NSAIDs | BASDAI, BASFI, VAS, adverse events | 24 weeks |
| Pang et al. 2020 [4] | Iguratimod 25mg Bid + Etanercept 25mg tiwce a week | Etanercept 25mg tiwce a week | ESR, CRP, BASDAI | 12 weeks |
| Qiu et al. 2016 [5] | Iguratimod 25mg Bid | NSAIDs+DMARDs | ESR, BASDAI, BASFI, VAS, back pain score, adverse events | 24 weeks |
| Xu et al. 2019 [6] | Iguratimod 25mg Bid + Celecoxib 0.2 g Qd. | Sulfasalazine 1 g Bid. + Celecoxib 0.2 g Qd. | BASDAI, BASFI, VAS, ESR, CRP, adverse events | 24 weeks |
| Yan et al. 2021 [7] | Iguratimod 50mg Qd + NSAIDs | NSAIDs + Placebo | BASDAI, BASFI, CRP, ESR, adverse events | 24 weeks |
| Yuan et al. 2020 [8] | Iguratimod 25mg Bid + Etoricoxib tablets 60 mg Qd. + ibuprofen 300 mg Tid. + methotrexate 15 mg once a week | Etoricoxib tablets 60 mg Qd. + ibuprofen 300 mg Tid. + methotrexate 15 mg once a week | VAS, CRP, ESR, adverse events | 12 weeks |
| Zeng et al 2016 [9] | Iguratimod 25mg Bid + Meloxicam 7.5 mg Qd. | Sulfasalazine 0.75 g Tid. + Meloxicam 7.5 mg Qd. | BASDAI, CRP, adverse events | 24 weeks |

**Reference**

1. Bai YJ, Wang XY, Yao YJ. Observation on the clinical effect of iguratimod in the treatment of axial spondyloarthritis[J]. Chinese Medical Innovation,2021,18(2):44-47. DOI:10.3969/j.issn. 1674-4985.2021.02.011.
2. Li X, Pan T, Chen MP. Observation of the curative effect of Iramod in the treatment of ankylosing spondylitis. J Med Theor and Prac, 2021, 34(17): 3009-3011.
3. Lin YP, Liu H, Gao JT(2019). Preliminary observation on the treatment of ankylosing spondylitis with Iramod. Journal of Clinical Rational Use, 012(014):9-13.
4. Pang LX, Zheng ZH, Li ZQ, et al(2020). Efficacy of Iramud combined with Etanercept in the treatment of ankylosing spondylitis. Journal of Tropical Medicine, 20(04):118-121.
5. Qiu YY Tang Y, Rui JB, Li J(2016). Observation on the clinical effect of Iramod in the treatment of refractory ankylosing spondylitis.Journal of Jiangsu University (Medical Edition), 26(03):235-239.
6. Xu BJ, Mo SQ, Xue XQ Wu Y(2019). Study on the efficacy and safety of Iramod in the treatment of ankylosing spondylitis. New Medicine, 50(12): 915-918.
7. Li Y, Li K, Zhao Z, Wang Y, Jin J, Guo J, Zhang J, Zhang J, Zhu J, Huang F. Randomised, Double-Blind, Placebo-Controlled Study of Iguratimod in the Treatment of Active Spondyloarthritis. Front Med (Lausanne). 2021 Jun 2;8:678864. doi: 10.3389/fmed.2021.678864. PMID: 34150809; PMCID: PMC8208078.
8. Yuan FF, Chen YH, Lin JX, Luo J(2020). Efficacy of islammod combined with methotrexate in the treatment of refractory ankylosing spondylitis and its effect on serum SOD and CTX-I levels in patients. Journal of Pharmaceutical Epidemiology,,29(03):163-165+205.
9. Zeng HQ, Kong WH, Zhuang P, Dong HJ, Yin ZH, Chen Xinpeng, Fu ZC, Ye ZZ(2016). Observation on the efficacy of Iramod in the treatment of ankylosing spondylitis.Hainan Medicine, 27(01):118-120.
